# Diversity and assembly patterns of mangrove rhizosphere mycobiome along the Coast of Gazi Bay and Mida Creek in Kenya

**DOI:** 10.1371/journal.pone.0298237

**Published:** 2024-04-18

**Authors:** Edith M. Muwawa, Huxley M. Makonde, Chinedu C. Obieze, Isabelle G. de Oliveira, Joyce M. Jefwa, James H. P. Kahindi, Damase P. Khasa

**Affiliations:** 1 Department of Biological Sciences, Pwani University, Kilifi, Kenya; 2 Centre for Forest Research and Institute for Systems and Integrative Biology, Université Laval, Québec, QC, Canada; 3 Department of Pure & Applied Sciences, Technical University of Mombasa, Mombasa, Kenya; 4 Laboratory of Mycorrhizal Associations, Department of Microbiology/BIOAGRO, Universidade Federal de Vicosa, Vicosa-MG, Brazil; Universite Libre de Bruxelles, BELGIUM

## Abstract

Fungi are among key actors in the biogeochemical processes occurring in mangrove ecosystems. In this study, we investigated the changes of fungal communities in selected mangrove species by exploring differences in diversity, structure and the degree of ecological rearrangement occurring within the rhizospheres of four mangrove species (*Sonneratia alba*, *Rhizophora mucronata*, *Ceriops tagal* and *Avicennia marina*) at Gazi Bay and Mida Creek in Kenya. Alpha diversity investigation revealed that there were no significant differences in species diversity between the same mangrove species in the different sites. Rather, significant differences were observed in fungal richness for some of the mangrove species. Chemical parameters of the mangrove sediment significantly correlated with fungal alpha diversity and inversely with richness. The fungal community structure was significantly differentiated by mangrove species, geographical location and chemical parameters. Taxonomic analysis revealed that 96% of the amplicon sequence variants belonged to the Phylum *Ascomycota*, followed by *Basidiomycota* (3%). Predictive FUNGuild and co-occurrence network analysis revealed that the fungal communities in Gazi Bay were metabolically more diverse compared to those of Mida Creek. Overall, our results demonstrate that anthropogenic activities influenced fungal richness, community assembly and their potential ecological functions in the mangrove ecosystems investigated.

## Introduction

Mangrove forests are regarded as blue carbon reservoir because they contribute approximately 10–15% of global carbon storage [1, 2]. In tropical and subtropical regions, mangroves are widespread and occupy more than a quarter of the global tropical coastline, where they serve as a barrier from strong waves and storms [2, 3]. Microorganisms are a major component of mangrove biodiversity, and the characteristic high levels of salinity, organic matter content and redox potential within mangroves provide an opportunity for the proliferation of specialized group of microorganisms involved in critical biogeochemical processes. Considering the ecological importance of mangroves and the role played by microorganisms thereof, it is important to understand the pattern of microbial community assembly in the face of increasing climate change. The mangrove ecosystem was chosen for this study because it is constantly affected by fluctuating events in terms of tidal variations, pH, temperature, salinity and nutrient availability [4]. Also, it uniquely lies at the land-water interface and thus is composed of microbiota from terrestrial soil, freshwater and the marine environment [5]. This makes the mangrove ecosystem an excellent habitat for study in terms of microbial diversity and their different functional roles.

Fungi are a diverse group of organisms composed of seven phyla which include *Basidiomycota*, *Ascomycota*, *Glomeromycota*, *Microsporidia*, *Blastocladiomycota*, *Neocallimastigomycota and Chytridiomycota* [6]. Fungal communities have been known to colonize diverse habitats such as tropical regions, extreme environments such as deserts, areas with high salt concentrations, ionizing radiation, deep-sea sediments and ocean hydrothermal areas [7]. Most fungi grow in terrestrial environments, though several species live partly or solely in aquatic habitats [8]. In most ecosystems, fungi are the major decomposers, playing an essential role in nutrient cycling as saprotrophs and symbionts that degrade organic matter into inorganic molecules, thereby contributing in ecological and biogeochemical processes [9]. In mangroves, fungi and bacteria alone constitute approximately 91% of the total biomass [10] and are known to significantly contribute to the breakdown of mangrove derived organic matter, therefore representing important food source for benthic fauna [11]. Also, fungal communities are known to co-evolve with mangrove plant species and contribute to nutrient uptake, productivity and resistance to environmental stressors [6]. Additionally, fungi play a role in microbial network interactions in various ecosystems including the mangrove sediments as portrayed by Booth and some others [12]. They are also thought to play a significant role in the ecology of mangrove forests and can be bio indicators of pollutants [13].

Significant efforts have been made to understand the mechanisms of microbiome assembly in mangroves but mainly focused on bacterial communities [2, 4, 14, 15]. Also, studies on fungal diversity in the mangrove ecosystem have mainly focused on taxonomic diversity of saprophytic fungi retrieved from intertidal, floating or immersed, pieces of trees and wood debris [3]. Information on fungal diversity in mangroves especially in the Kenyan mangroves is limited and poorly investigated. Mostly, it is based on culture-dependent assessments [16], which only accounts for a small percentage of fungi present in the environment [17]. Culture-independent techniques are therefore emerging as potential tools to ensure holistic insight into environmental systems, hence leading to an in-depth understanding of the complex mechanisms in an ecosystem [18]. For instance, studies employing high-throughput sequencing has revealed that the assembly of microorganisms can be explained by either niche theory (considers that microbial community assembly is determined by abiotic and biotic factors) or neutral theory which considers that stochastic factors including dispersal events control microbiome assembly in different environments [2, 19]. Also, microbial co-occurrence has been determined using high-throughput sequencing data in different environments [19–21].

Human activities through anthropogenic degradation of mangrove forests and introduction of contaminants like hydrocarbons, human wastewater, sewage, micro plastics and others have also led to the accumulation of artificially introduced nutrients in the mangrove ecosystems. These have altered the mangrove ecosystem equilibrium of the microbial community and its mangrove host [22].

Considering that mangrove species, human activities and environmental factors can influence the diversity and assembly of microbes in an ecosystem, we designed this study to investigate the following: (1) the overall fungal diversity associated with the rhizosphere of four mangrove species at Gazi Bay (a pristine mangrove site) and Mida Creek (a polluted mangrove site) in Kenya (2) the influence of differences in physicochemical parameters on mycobiome assembly in both geolocations and (3) the degree of ecological rearrangement and potential functional response of the fungal communities as a result of the pollution event at Mida Creek.

## Materials and methods

### Ethical statement

The National Commission for Science, Technology and Innovation of Kenya (NACOSTI) approved this study, the National Environmental Management Authority of Kenya (NEMA) provided the access permit (for field sampling), Kenya Wildlife Services (KWS) and Kenya Plant Health Inspectorate Services (KEPHIS) provided permits that facilitated the shipment of samples to Canada for physicochemical and environmental DNA analyses. The field study neither involved endangered nor protected species [15].

### Description of study site

We investigated two study sites: Mida Creek and Gazi Bay in Kenya. Mida Creek (03°34′S, 039°96′E), located in Kilifi County is about 88 Km North of Mombasa and approximately 25 km South of Malindi town in a planigraphic area of 32 km^2^ [23]. It has an average annual temperature of 27°C and characterized by a hot and humid tropical climate. Humidity is high throughout the year, up to 90% relative humidity during the rainy season [23]. In addition, the Creek is affected by anthropogenic degradation such as overharvesting of mangroves for firewood, timber and fish traps, pollution from plastics, faeces and oil spills, clearing of mangrove and conversion to other land uses such as aquaculture, urban development and tourism [24].

Gazi Bay is in Kwale County (4°44′S, 39°51′E), approximately 55Km from Mombasa, South Coast of Kenya. The Bay is sheltered from strong waves by the presence of the Chale peninsula to the East and a fringing coral reef to the South. The climate is hot and humid, and the average annual temperature and humidity are about 28°C and up to 95%, respectively [23]. The mangrove forests in the two studied sites display almost same zonation pattern among the dominant species (contribute over 80% of the mangrove formation in the sites with the estimated species contribution being; *A*. *marina* (30%), *R*. *mucronata* (25%), *S*. *alba* (15%) and *C*. *tagal* (10%)): *S*. *alba* (about 6–10 m tall) forms the outermost zone (seaward side) towards the open water followed by pure stands of *R*. *mucronata* (about 8–12 m tall) or mixed stands of *R*. *mucronata* and *Bruguiera gymnorrhiza* (about 10–20 m tall) and in turn these stands are followed by pure stands of *C*. *tagal* (about 3–5 m tall) and *A*. *marina* (about 12–18 m tall) along the Creek, as described by Matthijs et al. [25] and others [26, 27]. *R*. *mucronata* has well-developed prop roots that accumulate large stocks of debris, perhaps contributing to some accretion that supports the extensive tidal flats seen in the area [26].

### Collection of samples

Sampling was conducted according to the described methods by Wu and some others [28]. Four species of mangrove trees namely *S*. *alba*, *R*. *mucronata*, *C*. *tagal* and *A*. *marina* common to the two sites were selected. Four mangrove trees of each species at intervals of 10 m were chosen. For each individual mangrove species, the sediments (~100 g) were sampled vertically along the base of the plant at two depths (1–5 cm and 10–15 cm), using a standardized core sampler [29]. Eight samples (Four samples from each of the two sampling depths) were collected from each of the four-mangrove species in Gazi Bay and Mida Creek (making a total of 64 samples all together). The samples were transferred to Pwani University, Kenya, and maintained at -20°C, before their subsequent transfer to Université Laval, Canada, for further processing and analysis. All transfer of the samples was done using dry ice.

### Physicochemical analyses of soil samples

Analyses of soil samples for nitrogen, carbon, phosphorus, potassium, calcium, magnesium and sodium were conducted according to standard methods as described by Brupbacher et al. [30]. Determination of pH and electrical conductivity was done using the calcium chloride method at a ratio of 1:2 using a digital pH meter (Corning pH meter 140, Corning, New York) and electrical conductivity meter (Conductivity meter type CDM 2d radiometer Copenhagen) respectively.

### Total community DNA extraction, PCR protocol and Illumina MiSeq sequencing

Total genomic DNA was extracted directly from 0.25 g of soil using Power Soil DNA isolation kit (DNeasy PowerSoil Kit, Qiagen, Germany) in accordance with the manufacturer’s protocol. The extracted DNA was quantified using NanoDrop™ 2000 spectrophotometer (Thermo Fisher Scientific, MA, USA). Amplification of the fungal ITS2 gene, equimolar pooling and sequencing was performed at IBIS/Université Laval Plate-forme d’Analyses Génomiques (Québec, Canada). Briefly, amplification of the ITS regions was performed using the ITS3 mix primers and ITS4ngs sequence specific regions described by Tedersoo et al. [31] using a two-step dual-indexed PCR approach specifically designed for Illumina instruments.

In a first step, the gene specific sequence was fused to the Illumina TruSeq sequencing primers and PCR was carried out in a total volume of 25 μL that contains 1X Q5 buffer (NEB), 0.25 μM of each primer, 200 μM of each dNTPs, 1 U of Q5 High-Fidelity DNA polymerase (NEB) and 1 μL of template cDNA. The PCR started with an initial denaturation at 98°C for 30 s followed by 35 cycles of denaturation at 98°C for 10 s, annealing at 55°C for 10 s, extension at 72°C for 30s and a final extension at 72°C for 2 min. The PCR reaction was purified using the Axygen PCR cleanup kit (Axygen, Corning, Arizona, USA). Quality of the purified PCR products was checked on a 1% agarose gel. Fifty to 100-fold dilution of this purified product was used as a template for a second PCR step with the goal of adding barcodes (dual-indexed) and missing sequence required for Illumina sequencing. Cycling for the second PCR were identical to the first PCR but with 12 cycles. PCR reactions were purified as above, checked for quality on a DNA7500 Bioanlayzer chip (Agilent Technologies) and then quantified spectrophotometrically with the NanoDrop ND-1000 spectrophotometer (Marshall Scientific). Barcoded Amplicons were pooled in equimolar concentration for sequencing on the illumina Miseq. The following oligonucleotide sequences were used for amplification:

ITS3tagmix1: ACACTCTTTCCCTACACGACGCTCTTCCGATCTTAGACTCGTCATCGATGAAGAACGCAG

ITS3tagmix2: ACACTCTTTCCCTACACGACGCTCTTCCGATCTTAGACTCGTCAACGATGAAGAACGCAG

ITS3tagmix3: ACACTCTTTCCCTACACGACGCTCTTCCGATCTTAGACTCGTCACCGATGAAGAACGCAG

ITS3tagmix4: ACACTCTTTCCCTACACGACGCTCTTCCGATCTTAGACTCGTCATCGATGAAGAACGTAG

ITS3tagmix5: ACACTCTTTCCCTACACGACGCTCTTCCGATCTTAGACTCGTCATCGATGAAGAACGTGG

ITS4ngs: GTGACTGGAGTTCAGACGTGTGCTCTTCCGATCTTTCCTSCGCTTATTGATATG generic forward second-PCR primer AATGATACGGCGACCACCGAGATCTACAC[index1]ACACTCTTTCCCTACACGC and generic reverse second-PCR primer CAAGCAGAAGACGGCATACGAGAT[index2]GTGACTGGAGTTCAGACGTGT. The primers used in this work contain Illumina specific sequences protected by intellectual property (Oligonucleotide sequences © 2007–2013 Illumina, Inc. (All rights reserved).

### Sequence processing

Demultiplexed paired-end sequences obtained from the sequencing centre were processed using QIIME2 [32]. Sequences were first quality checked and the information derived thereof was used to trim low quality ends of the sequence reads. The filtering of marginal sequencing errors, chimeric sequences and clustering of high-quality reads into amplicon sequence variants (ASVs) was achieved using DADA2 [33]. Classification of the representative sequences for each ASV was done against the UNITE database using VSEARCH consensus classifier. The ASV count table was rarefied to an even sampling depth and singletons removed prior to diversity analyses.

### Statistical analyses

All statistical analyses were performed using R v3.6.3 [34]. The non-parametric Kruskal-Wallis H test and Fisher’s least significance difference (LSD) post hoc analysis in the *agricolae* package v1.3–2 [35] was used to determine significant differences in soil physicochemical properties.

Linear discriminate analysis (LDA) effect size (LEfSe) [36] and Random Forest analysis [37] as implemented in *MicrobiomeAnalyst* [38], was used to test for differences in taxonomic composition across sites and mangrove species. Fungal phylotypes having an LDA score *≥*3 and adjusted p-value ≤0.05 were differentially abundant. Alpha diversity was based on richness (observed ASVs), Shannon entropy and Pielou’s evenness. Determination of fungal community compositional and structural differences across sites, mangrove species and sampling depth was based on Bray-Curtis distance. Permutational multivariate analysis of variance (PERMANOVA) was used to test for differences across sites and mangrove species. All diversity analyses were performed using QIIME2. The influence of some environmental factors on community level differentiation was determined by constrained redundancy analysis (RDA). RDA was performed on Hellinger transformed ASV-abundance table using the “amp_ordinate()” function in *ampvis2* R package. The significance of the RDA model was tested using the vegan function “anova.cca”. To determine the potential functions of the fungal communities, their functional guilds were predicted using FUNGuild [39].

Furthermore, the importance of fungal phylotypes and environmental factors on the assembly of the different mycobiomes was determined by constructing co-occurrence networks using Conet [40]. Prior to network generation, the dataset of fungal abundance was compositionally corrected using the centred log-ratio (*clr*) approach. Also, to reduce the network complexity, the dataset was filtered to remove phylotypes occurring in less than 50% of the samples for each mangrove specie. The networks generated were thereafter visualized using Gephi (https://gephi.org/). To identify potential fungal communities, the networks were clustered into modules (communities of fungi and associated chemical properties) using a multi-level modularity optimization algorithm [41]. To determine the importance of the fungal phylotypes and environmental variables on the community assembly, the connectivity of each node was determined by calculating the within module connectivity (Zi) and among module connectivity (Pi). Thereafter, the nodes were sorted into four sub-categories (a) peripheral nodes, (b) connectors, (c) module hubs and (d) network hubs, as described by Guimera and Amaral [42].

## Results

### Physicochemical factors across mangrove species and sites

Sediment physicochemical parameters revealed significant differences both across sites and among mangrove species (Table 1). Mean values of the analysed physicochemical parameters were generally higher in Mida Creek samples compared to samples obtained from Gazi Bay. Calcium and pH were two physicochemical factors that were significantly higher (*p ≤ 0*.*001*) in all rhizospheres of Mida Creek mangrove species. Exceptions included potassium, magnesium, sodium, phosphorus, carbon, nitrogen, EC and salinity that were all significantly higher in the rhizosphere of *R*. *mucronata* in Gazi Bay.

**Table 1:** Mean values of physicochemical parameters for the different mangrove species across both Mida Creek and Gazi Bay.

Physicochemical Parameters	*A*. *marina*		*C*. *tagal*		*R*. *mucronata*		*S*. *alba*	
Site	Gazi	Mida	Gazi	Mida	Gazi	Mida	Gazi	Mida
Calcium (mg/kg)	325.75± 64.73	14085.5± 3686.55***	168.25± 27.76	79312.88±38869.51***	2670.62±544.23	62731.12±26956.45***	477± 147.65	52097± 18982.24***
Potassium (mg/kg)	663.12± 192.89	494±182.67	178.25± 40.78	464± 111.51***	1588.87±172.27***	597.375 ± 211.89	461.87± 138.12	594.75 ± 82.25*
Magnesium (mg/kg)	379.125± 76.07	841.75± 470.97*	115.12 ± 21.66	1776± 660.54***	1856.12±140.19*	1307.12 ± 665.24	406.87± 68.52	1166.62± 345.31***
Sodium (mg/kg)	2369±815.28	4162±3483.99	155.87±40.50	3933.87±1448.40***	8472.25±967.11***	3263.75±1446.15	1978±431.28	2948.37±471.96***
Phosphorus (mg/kg)	55±16.29	136.12±33.09***	40±2.87	164.5±61.01***	187.75±49.67**	98.87±54.49	55.125±14.06	99.75±36.32**
Total carbon (mg/kg)	0.82±0.41	1.90±0.57***	0.26±0.07	4.55±1.56***	7.40±0.85***	2.69±1.67	0.99±0.23	2.25±0.60***
Nitrogen (mg/kg)	0.04±0.02	0.16±0.03***	0.01±0.01	0.18±0.04***	0.37±0.06***	0.11±0.05	0.07±0.01	0.09±0.01*
Electrical conductivity (S/m)	5.78±0.95	4.56±1.39	3.37±1.31	6.51±2.98*	12.37±1.00***	4.05±1.36	4.88±0.88	5.22±1.34
pH	7.05±0.55	8.17±0.21***	6.21±0.10	7.93±0.07***	6.09±0.15	8.08±0.28***	6.08±0.08	7.91±0.16***
Salinity (mg/kg)	3.13±0.55	2.43±0.79	1.77±0.72	3.58±1.75*	7.08±0.61***	2.15±0.78	2.61±0.50	2.81±0.77

Values represent mean and standard deviation. Superscripts beside values are significantly different measures (*p ≤ 0*.*05*) based on Fisher’s least significance difference. (Significance codes

‘***’ 0.001

‘**’ 0.01

‘*’ 0.05)

Overall comparison of physicochemical factors based on mangrove species across both study sites revealed that potassium, magnesium, sodium, carbon, nitrogen (*p ≤ 0*.*001*), EC, salinity (*p* ≤ *0*.*01*) and phosphorus (*p* ≤ *0*.*05*) were all significantly higher in the rhizosphere of *R*. *mucronata*. Also, overall site comparison revealed pH, calcium, magnesium (*p* ≤ *0*.*001*) and phosphorus (*p* ≤ *0*.*01*) were all significantly higher in Mida Creek while EC and salinity were significantly higher (*p ≤ 0*.*05*) in Gazi Bay samples. Across depths (1–5 cm and 10–15 cm), the differences in physicochemical parameters were also significant on comparison of the two sites (S1 Fig); however, within site comparisons revealed that vertical differences in physicochemical parameters were not significant (*p* > *0*.*05*), except for Calcium, which was significantly higher at the surface than subsurface in Mida creek (S2 and S3 Figs).

### Alpha diversity of mangrove mycobiome

A total of 3,406,382 sequence reads from the 64 mangroves rhizosphere samples were quality-filtered and 317,851 high-quality ITS sequence reads clustered into 1,022 fungal ASVs. Five hundred and fifty-one ASVs were unique to Gazi Bay mangrove rhizospheres, 321 were unique to Mida Creek, while 150 ASVs were found in both Gazi Bay and Mida Creek (S4 Fig). Based on mangrove species differentiation, *C*. *tagal* had the highest number of unique ASVs in Gazi Bay, whereas *A*. *marina* had more unique ASVs than any other mangrove species in Mida creek (S5 Fig). Both mangrove species were more distributed in the outermost section of the two sites, away from the open water. Also, regardless of geographic and chemical differences, ASVs ranging from 19–41 were present within the rhizosphere of similar mangrove species found in both Gazi Bay and Mida creek (S6 Fig).

**Fig 1:**
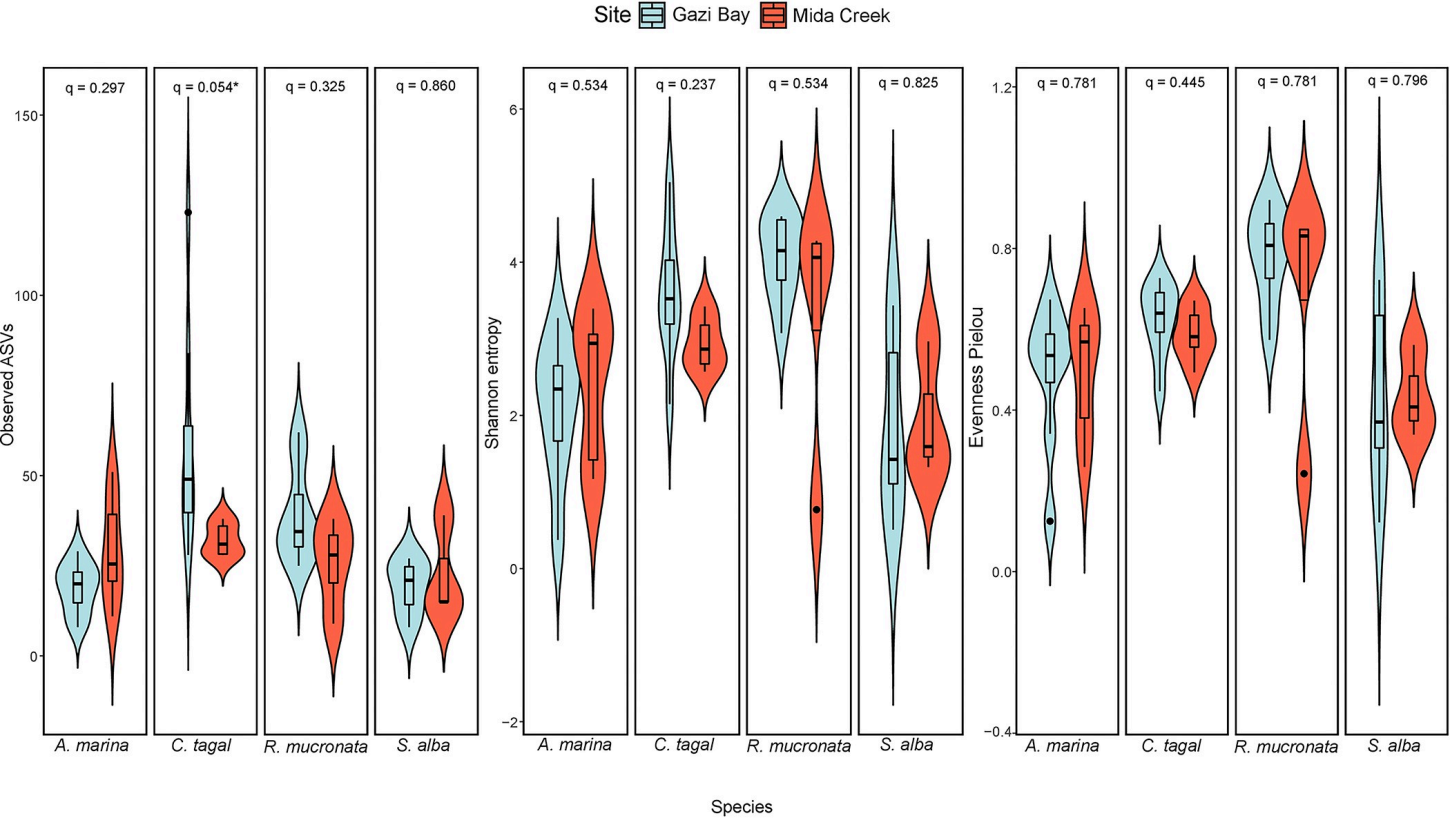
Alpha diversity comparison based on mangrove species and site differentiation.

Investigation of species distribution (Pielou’s evenness) and Shannon’s entropy revealed that there were no significant differences (*p > 0*.*05*) in fungal species diversity between similar mangrove species in Gazi Bay and Mida Creek (Fig 1). However, fungal richness (Observed ASVs) was significantly higher in Gazi Bay than Mida Creek, for *C*. *tagal* mangrove species. Further mangrove species-based comparison in the individual sites revealed that, in Gazi Bay, fungal richness was significantly higher in the rhizosphere of both *C*. *tagal* and *R*. *mucronata*, while in Mida creek, the differences among mangrove species were not significant (*p* > 0.05). Similar observations were made for Shannon’s entropy and Pielou’s evenness, with slight differences in the level of significance, as presented in S1 Table.

Spearman’s rank correlation analysis revealed that Shannon’s entropy correlated with phosphorus (r = 0.27, *p* = 0.05); Pielou’s evenness correlated with nitrogen (r = 0.29, *p* = 0.04), carbon (r = 0.32, *p* = 0.02) and phosphorus (r = 0.30, *p* = 0.03), while observed ASVs inversely correlated with potassium (r = -0.28, *p* = 0.05).

### Community differentiation and influence of environmental factors

**Fig 2:**
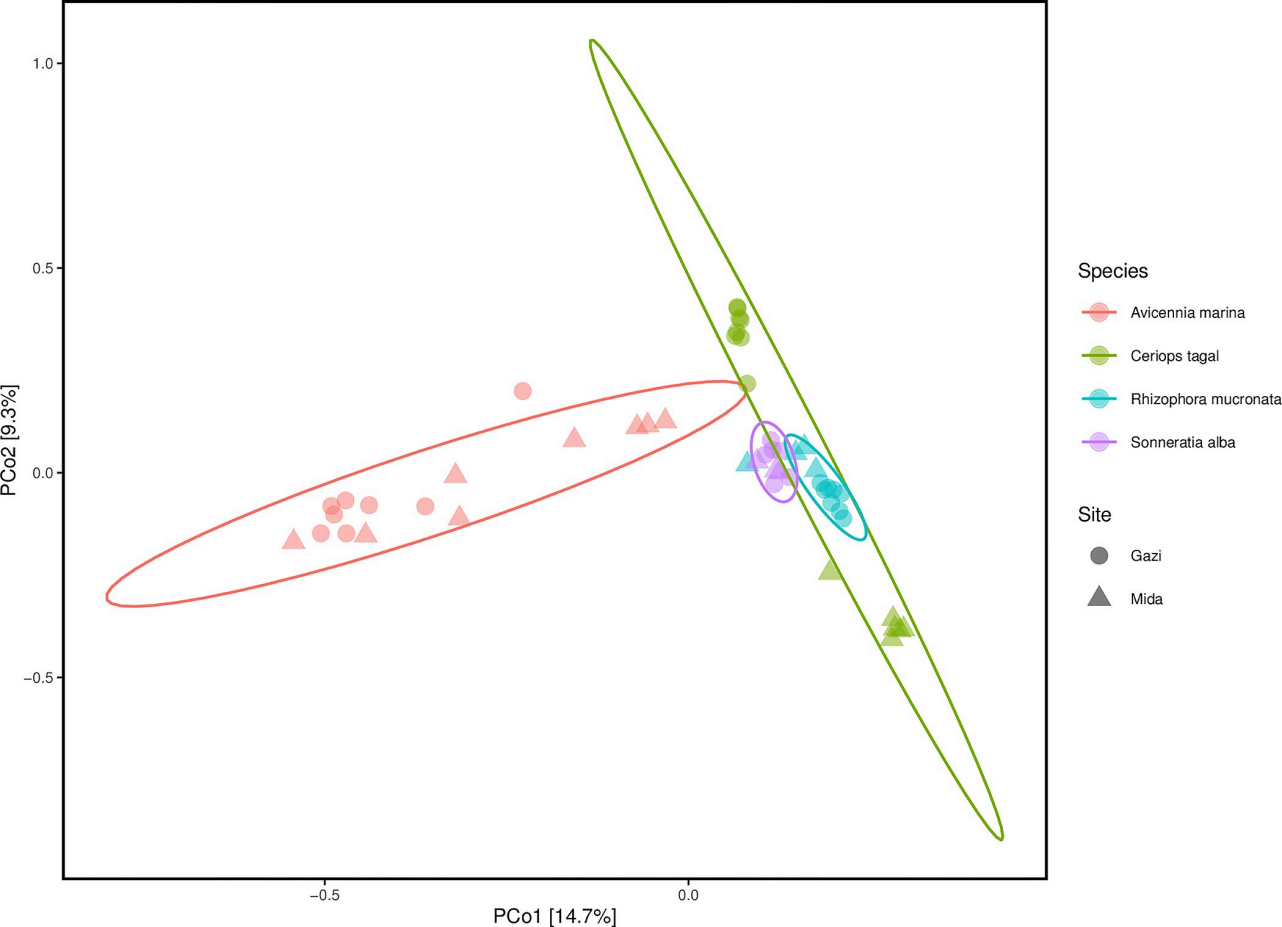
Fungal community composition and structure in the mangrove rhizosphere sediments based on Bray-Curtis dissimilarities.

Structural differentiation based on Bray-Curtis distance revealed appreciable fungal compositional differences between sites and among mangrove species (Fig 2). Close associations were observed between the fungal communities in the rhizosphere of *R*. *mucronata* and *S*. *alba*, while the fungal community composition and structure in the rhizosphere of *A*. *marina* were observed to be distinct. Overall, samples mostly separated according to mangrove species, followed by site differences. Accordingly, permutational multivariate analysis of variance (PERMANOVA), which was based on a nested or hierarchical model, where mangrove species was nested under sites, revealed that a higher proportion of the observed variation (~35%) was explained by the differences in mangrove species (PERMANOVA R^2^ = 0.348, *p* < 0.001), while site differences explained only ~5% of the observed variation (PERMANOVA R^2^ = 0.048, *p* < 0.001). Further pair-wise comparison revealed that the fungal community compositions were significantly different for similar mangrove species in different sites, and among different mangrove species of the same site, except for *R*. *mucronata* and *S*. *alba*, which had similar communities in Mida creek (S2 Table).

**Fig 3:**
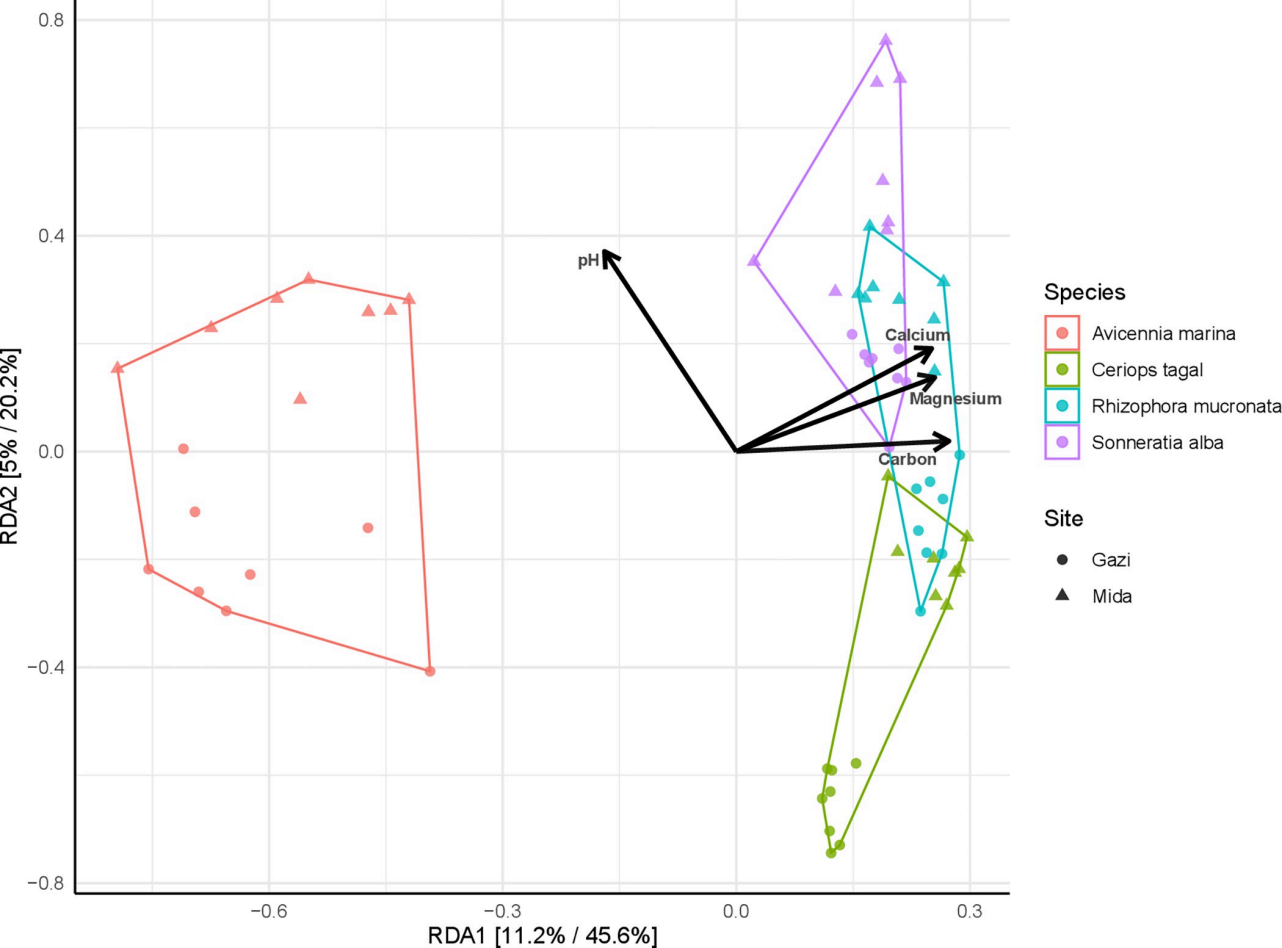
Constrained redundancy analysis showing contributions of environmental terms to fungal community composition.

The RDA model presented in Fig 3 was significant in explaining the influence of environmental variables on the fungal communities. Total variation explained by both mangrove species and site differences was 34% (variance = 0.345; F = 4.92; *p* = 0.001). More details on the variance explained by the constrained variables are presented in S3 Table. The significance of environmental factors that fitted into the RDA model revealed that calcium, magnesium, pH, phosphorus and carbon, were among the many chemical factors that significantly influenced the fungal community composition (Table 2) and contributed to the separation of the communities from both sites (Fig 3).

**Table 2:** Goodness-of-fit statistics (R^2^) for environmental terms fitted into the constrained RDA model.

Physicochemical parameters	RDA1	RDA2	r^2^	Pr(>r)
Calcium	0.73525	0.6778	0.2774	0.002**
Potassium	0.34799	0.9375	0.2258	0.002
Magnesium	0.55708	0.83046	0.616	0.001**
Sodium	0.2224	0.97495	0.3472	0.001
Phosphorus	0.36256	0.93196	0.5209	0.001
Carbon	0.59217	0.80581	0.622	0.001*
Nitrogen	0.43809	0.89893	0.4975	0.001
Electrical conductivity	0.44102	0.8975	0.3026	0.001
pH	-0.58133	0.81367	0.1515	0.008***
Salinity	0.45111	0.89247	0.3015	0.001

For the fungal phylotypes, Pearson’s correlation analysis indicated that calcium correlated with *Nectriopsis* (r = 0.41, p < 0.001); Magnesium correlated with *Aspergillus* (r = -0.31, p = 0.01), *Corollospora* (r = -0.24, p = 0.05), *Lasiodiplodia* (r = -0.26, p = 0.04), *Scedosporium* (r = 0.25, p = 0.05) and *Nectriopsis* (r = 0.32, p < 0.01); carbon correlated with *Ceriosporopsis* (r = 0.26, p = 0.04), *Trichoderma* (r = 0.24, p = 0.05) and *Scedosporium* (r = 0.38, p < 0.01), while pH correlated with *Lasiodiplodia* (r = -0.24, p = 0.05), *Penicillium* (r = 0.29, p = 0.02), *Trichoderma* (r = -0.31, p = 0.01), *Scedosporium* (r = -0.29, p = 0.02) and *Pseudopyricularia* (r = -0.24, p = 0.05).

### Fungal taxonomic composition

The most abundant fungal phyla across both study sites were Ascomycota (96%) and Basidiomycota (3%). Others less than 1% included, Chytridiomycota, Entomophthoromycota and Blastocladiomycota (S7 Fig). At the class level, *Sordariomycetes* (42%) was the most abundant across both sites, and this was followed by *Dothideomycetes* (31%), *Eurotiomycetes* (12%), *Rhizophydiomycetes* (2%) and *Agaricomycetes* (2%). The most abundant fungal families were *Aspergillaceae* (10%), *Halosphaeriaceae* (10%), *Cordycipitaceae* (6%) and *Lulworthiaceae* (3%). Details on other fungal classes and families can be found in S4 Table.

**Fig 4:**
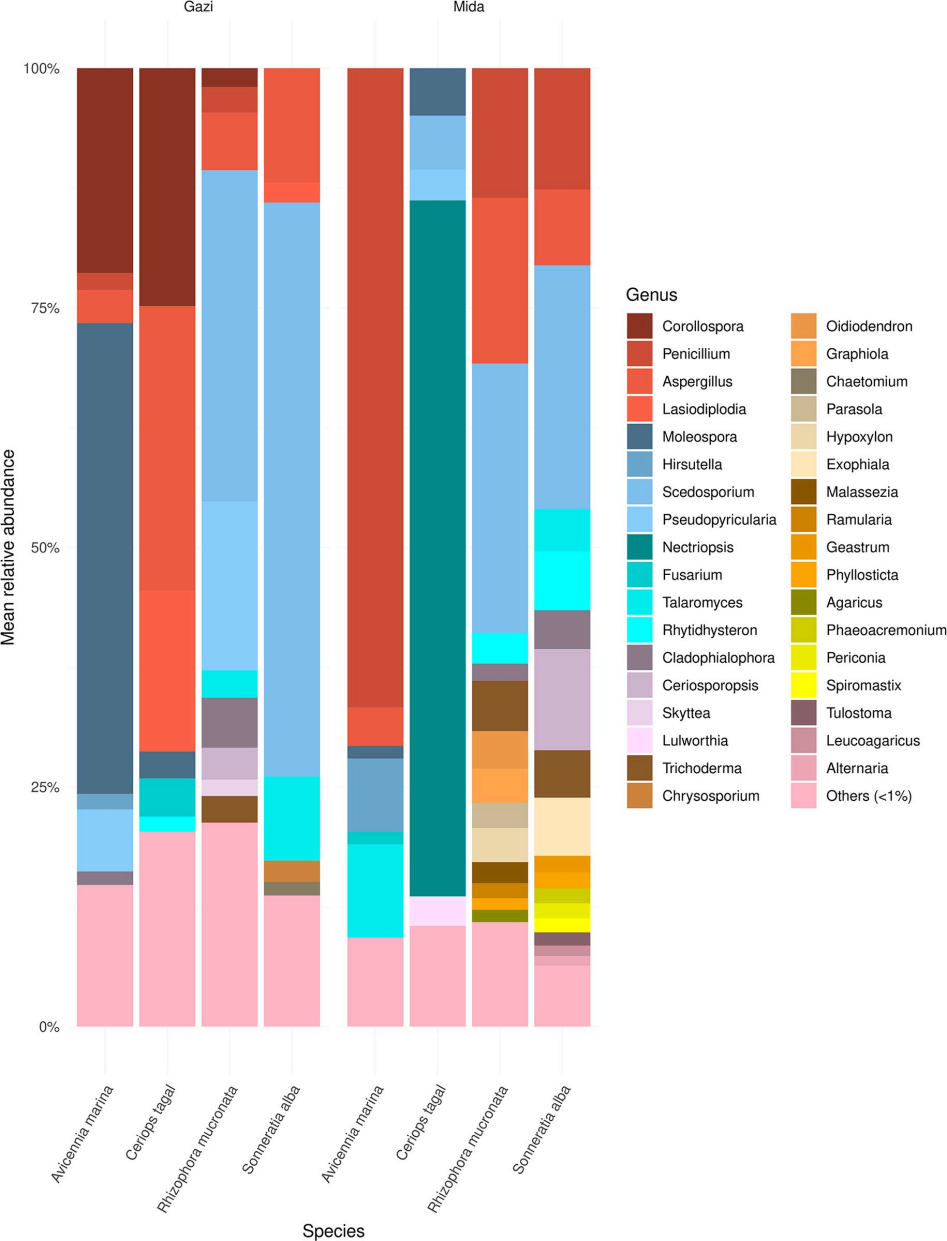
Fungal composition in the rhizosphere of mangrove species in both Gazi Bay and Mida Creek.

At the genus level taxonomic rank, 44% of the ASVs were successfully classified and those with relative abundance >1% in any of the mangrove species are presented in Fig 4. *Penicillium*, *Aspergillus*, *Corollospora*, *Scedosporium*, *Moleospora*, *Lasiodiplodia*, *Talaromyces* and *Nectriopsis* were among the dominant classified genera. The mangrove species with the most consistent taxonomic profile, regardless of site differences, were *R*. *mucronata* and *S*. *alba*, which saw a high abundance of *Scedosporium* and *Aspergillus*, whereas the taxonomic profile of *C*. *tagal* was particularly distinct, with *Nectriopsis*, dominating in Mida creek, while *Corollospora*, *Aspergillus* and *Lasiodiplodia* were dominant in Gazi bay. Determination of differentially abundant (*p* adjusted *≤* 0.05; LDA ≥ 3.0) fungal genera based on mangrove species differentiation revealed that *Nectriopsis* was differentially abundant in the rhizosphere of *C*. *tagal* in Mida Creek; *Moleospora* was differentially abundant in the rhizosphere of *A*. *marina* in Gazi Bay; *Penicillium* was differentially abundant in the rhizosphere of *A*. *marina* in Mida Creek; *Aspergillus*, *Scedosporium* and *Malassezia* were differentially abundant in the rhizosphere of *S*. *alba* in Gazi Bay; *Cladophialophora* was differentially abundant in the rhizosphere of *R*. *mucronata* in Gazi Bay, while *Hypoxylon* was differentially abundant in the rhizosphere of *R*. *mucronata* in Mida Creek (S8A Fig). Overall, *Nectriopsis*, *Hypoxylon* and *Penicillium* were among the differentially enriched genera in Mida Creek compared to Gazi Bay (S8B Fig).

### Community interaction and potential function

**Fig 5:**
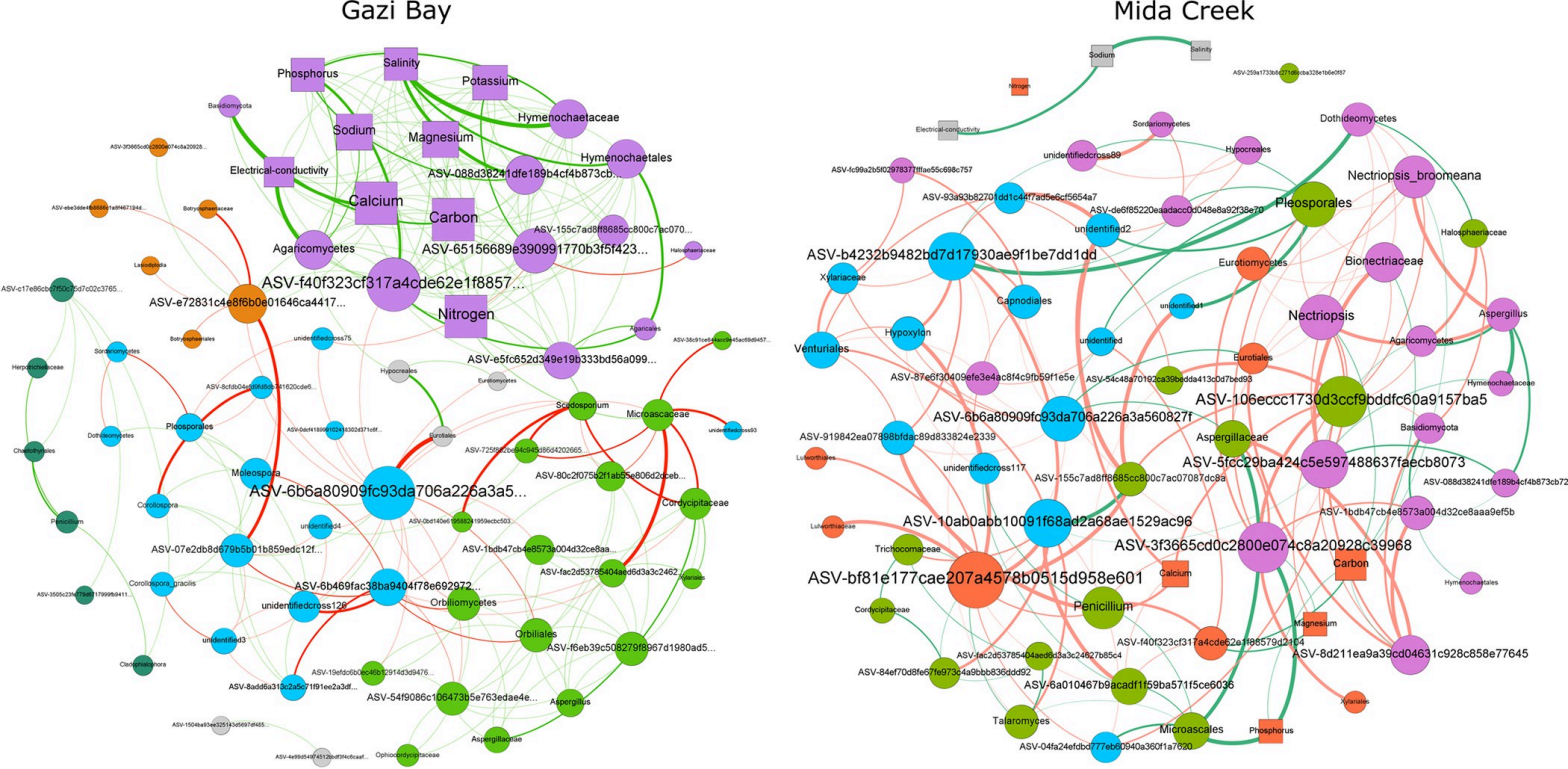
Co-occurrence network of fungal communities in Gazi Bay and Mida Creek. The round nodes represent fungal phylotypes (ASVs) while the square nodes are environmental variables. The size of each phylotype or environmental variable is proportional to their betweenness centrality. Nodes are coloured according to clusters or modules (communities) while nodes coloured grey have less than four interactions. The connections (edges) stand for significant (FDR-adjusted p<0.01) associations while their sizes are proportional to ρ. The green coloured edges indicate positive associations, while those coloured red represent inverse associations.

Fungal community interaction, including niche partitioning and the influence of chemical parameters was investigated using co-occurrence networks. Two networks, respectively representing potential interactions within mangrove rhizospheres in Gazi Bay and Mida Creek were constructed (Fig 5). Multiple network topological properties indicated that the co-occurrence pattern of the fungal communities in Gazi Bay differed markedly from those of Mida Creek (S5 Table). The main differences are the clustering coefficient, the average weighted degree and the proportions of positive and negative associations. Also, the network was more complex (connections between phylotypes and the total number of connected phylotypes) in Gazi Bay compared to Mida Creek.

**Fig 6:**
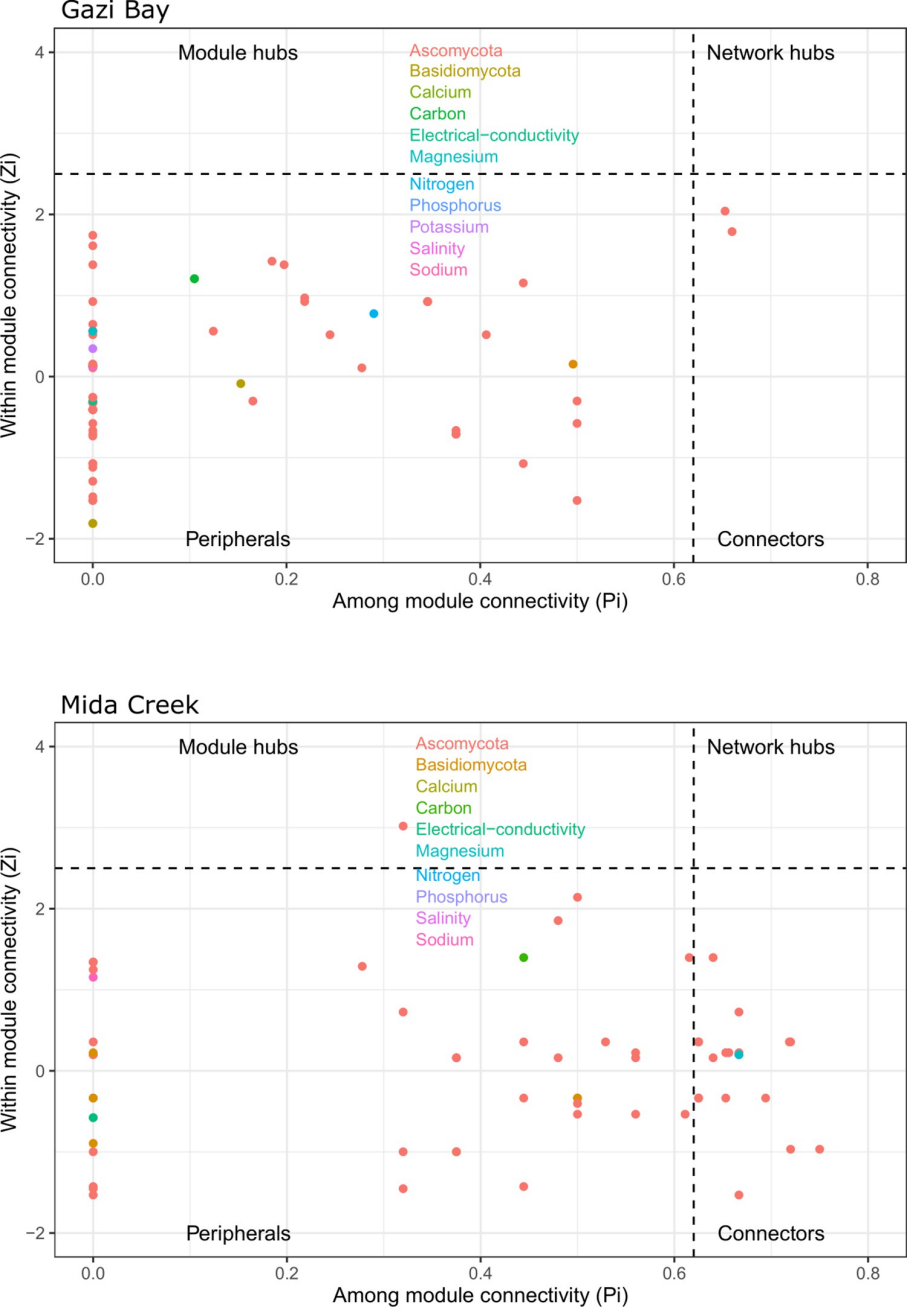
Node classification of the co-occurrence networks based on within module connectivity and among module connectivity criteria.

To identify fungal assemblages that potentially share a niche within the mangrove rhizospheres, the networks were clustered into modules (fungal communities or ecological clusters). Five modules were detected in Gazi Bay and 3 in Mida Creek. The module distribution pattern reflected fungal niche separation that appears to be based on the influence of the different mangrove species. For example, in Gazi Bay, *Corollospora* and *Moleospora*, which were dominant in the rhizosphere of *A*. *marina* were also part of the same ecological niche; *Scedosporium* and *Aspergillus*, which were dominant in the rhizosphere of both *R*. *mucronata* and *S*. *alba* also shared the same ecological niche. *Agaricomycetes*, *Sordariomycetes* and *Dothideomycetes* dominated most of the modules in both geographic locations. Also, some fungal phylotypes were associated with the sediment physicochemical parameters both in Gazi Bay and Mida Creek. While the species–physicochemical associations were mostly positive in Gazi Bay, this was not the case in Mida Creek, where several associations between fungal phylotypes and mangrove physicochemical parameters were antagonistic. Furthermore, in Gazi bay, only connectors belonging to the Ascomycota phylum were detected; there were no module hubs or network hubs, while in Mida Creek, an unidentified Capnodiales ASV was detected as a module hub. Also, chemical parameters, including phosphorus, magnesium and some phylotypes belonging to the Ascomycota phylum were detected as connectors (Fig 6).

**Fig 7:**
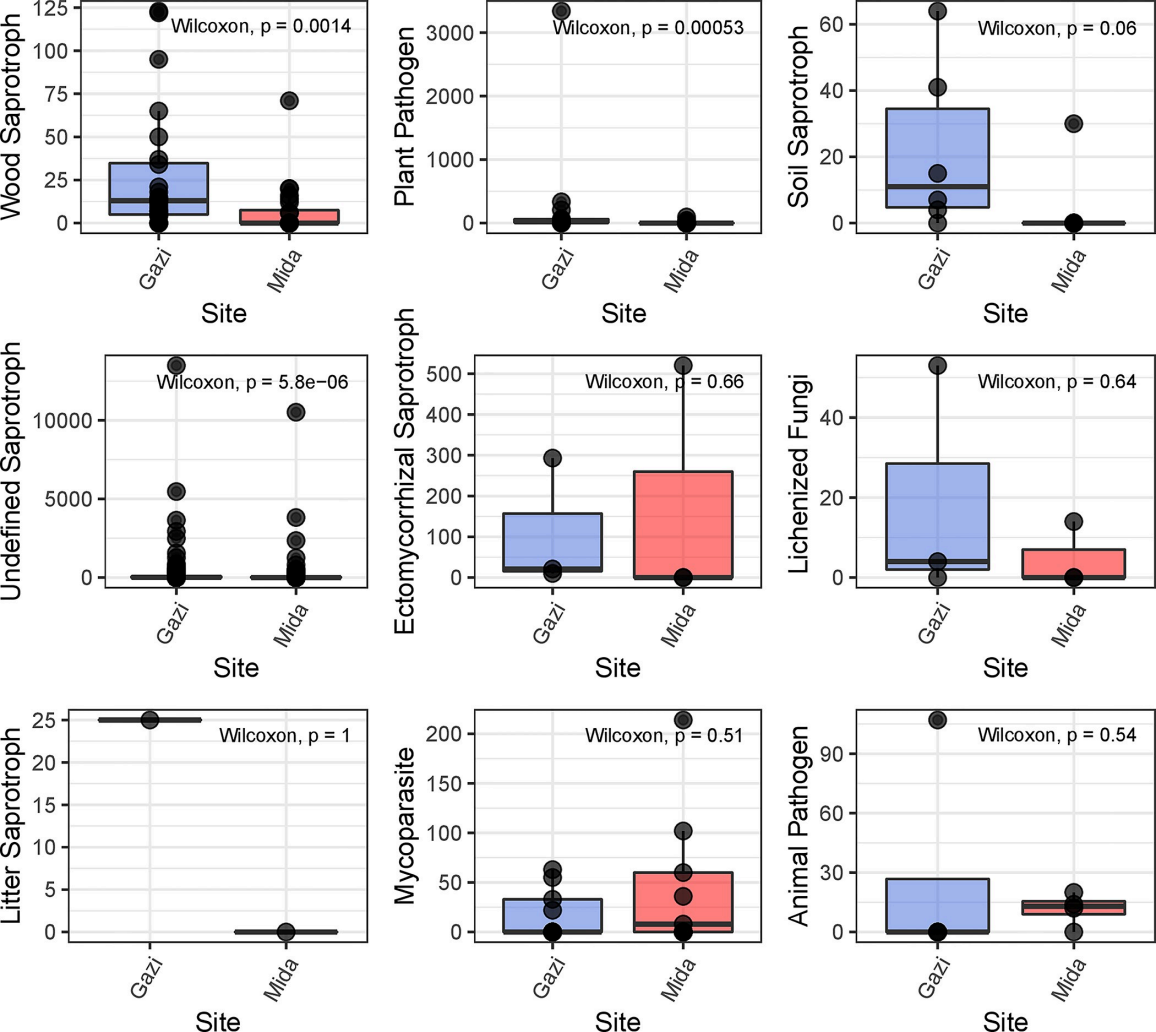
FUNGuild profile of mangrove rhizospheric mycobiome in Gazi bay and Mida Creek. *Gazi = Gazi Bay; Mida = Mida Creek.

Functional guilds prediction, to corroborate the observed differences in the co-occurrence network revealed that the proportion of wood saprotrophs, soil saprotrophs, undefined saprotroph, litter saprotrophs and lichenized fungi were higher in Gazi Bay, while the proportion of both mycoparasite and ectomycorrhizal saprotroph were higher in Mida Creek (Fig 7).

## Discussion

Fungi are important in soil as decomposers, and they play essential roles in many aspects of ecosystem development, function, and stability. Also, fungi are critical components of plant rhizosphere, where they contribute to plant nutrient uptake, pathogen resistance and energy cycling. Hence, understanding their diversity, community structure, function and assembly patterns is useful in underpinning the influence of anthropogenic activities on critical ecosystem functions. Accordingly, we employed the Illunima MiSeq sequencing technology to determine the influence of nutrient influx on fungal diversity, community composition and co-occurrence patterns in the rhizosphere of four mangrove species (*Sonneratia alba*, *Rhizophora mucronata*, *Ceriops tagal* and *Avicennia marina*), which are common along the Kenyan Coastline. Since plant tree species [28, 43] and geographic location [4, 44] also influence fungal communities, these factors were also investigated in this study.

Alpha diversity investigation revealed that both species distribution and Shannon’s entropy were not significantly different on comparison of the mangrove species in Gazi Bay and Mida Creek, while species richness was found to be significantly higher in some of the mangrove species in Gazi Bay (Fig 1). This observation is consistent with previous studies that investigated the effect of nutrients [45] and seasonal variation [46] on fungal alpha diversity in mangrove ecosystems. Both studies reported that fungal species diversity was relatively stable while species richness was influenced by differences in physicochemical factors. In the present study, fungal richness inversely correlated with potassium thus, demonstrating that it is an important determinant of fungal richness, within the rhizosphere of mangrove species, along the Kenyan coast. Further investigation of ASVs shared between same mangrove species in the different locations revealed that the outermost mangrove species (away from the open water) had a higher percentage of unique ASVs (S5 Fig). This observation suggests an influence from the surrounding watersheds, while the proportions of ASVs shared between same mangrove species in the different locations demonstrates the influence of mangrove species differentiation on the composition of associated ASVs. Collectively, these findings suggest that alpha diversity in the mangrove ecosystems were co-influenced by both physicochemical parameters and the selective pressure of host mangrove species. Though, our observation that there were no significant differences in alpha diversity among different mangrove species in Mida Creek, suggests that nutrient influx reduced the influence of mangrove species on fungal species selection.

Investigation of the fungal community structure revealed that the different mangrove species, site differences and physicochemical properties had a significant influence on the rhizosphere fungal communities. Specifically, the RDA model, constrained to plant species and site of sampling (Fig 3) was significant and respectively explained 20% and 5% of the total variation in fungal community composition. This finding corresponds to our observation on the bacterial communities of these mangrove species [15]. In both studies, the influence of plant selection mechanisms on the microbial communities was higher than site differences. Previous studies have demonstrated that below-ground microbial communities contribute to plant growth and survival through nutrient synthesis, mobilization and competition against soil-borne pathogens [47]. Thus, these benefits appear to moderate the relationship between plants and their associated microbial communities. Similarly, several studies have shown that geographic difference is an important factor that can influence plant-associated microbial communities [31, 48]. However, the influence of this factor largely depends on distance and on the differences in environmental parameters, including the soil texture, pH and available nutrients [49]. Accordingly, the RDA model revealed that the mangrove sediment chemical parameters contributed to the fungal community structure (Fig 3). The significance of environmental factors that fitted onto the RDA model revealed that calcium, magnesium, pH and carbon significantly influenced the fungal community structure and composition in the two sites. This finding is consistent with results from Purahong et al. [50], where they reported that physicochemical properties of the mangrove soil influenced the fungal community composition. Overall, this demonstrates a strong inter-relationship among environmental properties and fungal communities in the mangrove environment. However, the small proportion of the observed variance explained by these factors implies that a large percentage of the fungal community dissimilarities may be attributable to unaccounted deterministic processes, or to stochastic processes, including drift and species dispersal [49].

Taxonomic classification revealed five identified fungal phyla in both study sites (S7 Fig). The phylum *Ascomycota* was the most abundant in the rhizosphere of all mangrove species in the two sites, followed by the phylum *Basidiomycota*. The occurrence of the Dikarya as dominant phyla in this study is consistent with previous studies on mangrove ecosystems that pointed similarly to a prevalence of *Ascomycota* and *Basidiomycota* [6, 17]. Members of these two phyla have been reported to play important ecological functions in mangrove environments, including the utilization and recycling of nutrients [9]. Ascomycetes from marine environments are an important ecological assembly of saprophytic microbes occurring in different substrata rich in lignin, cellulose, or chitin [51]. Other trophic levels are dependent on the lignocellulose-cleaving capability of these fungi that allow this complex substrate to enter the food web [51]. Among the *Ascomycota*, members of the classes *Dothideomycetes*, *Sordariomycetes* and *Eurotiomycetes* were found to be dominant. On the other hand, members of *Agaricomycetes* and *Tremellomycetes* were found to be dominant among the *Basidiomycota*. The study by Simões et al. [6] also reported the same trend in their findings. Additionally, these classes have also been frequently reported to be found in large proportions in deep-sea sediments [52, 53], indicating that they are ubiquitous in the marine environment. The phyla *Chytridiomycota*, *Blastocladiomycota* and *Entomophthoromycota* were among the least observed in this study. Our findings are also consistent with those of Liu et al. [9] and Devadatha et al. [54], who found a smaller proportion of these phyla in their study.

The most abundant genera in both sites included *Scedosporium*, *Ceriosporopsis*, *Penicillium*, *Aspergillus* and *Talaromyces*, that have been detected and recovered mostly from the mangrove environment [50, 55, 56]. These genera have been demonstrated as a source of secondary metabolites from the mangrove environment [55]. Determination of differentially abundant phylotypes between the two sites and among the mangrove species revealed some significant differences in both Gazi Bay and Mida Creek. Among the differentially abundant phylotypes were *Nectriopsis*, *Moleospora*, *Scedosporium*, *Penicillium*, *Aspergillus*, *Cladophialophora* and *Hypoxylon*. These differences among similar mangrove species can be attributed to the variation in auto-correlating environmental and chemical factors. The differential abundance of *Hypoxylon* in the anthropogenically disturbed Mida creek is not surprising as some species of these genera are important plant symbionts and produce organic compounds that are active against several plant pathogens, including *Botrytis cinerea*, *Phytophthora cinnamomi* and *Cercospora beticola* [57]. Similarly, several species of *Penicillium* isolated from mangroves are important source of bioactive materials that are inhibitory to microbial pathogens [58]. Thus, the observed differential abundance of these genera in Mida creek is likely to support plant stability in response to environmental changes resulting from human disturbance.

The co-occurrence network enabled an evaluation of the degree of ecological rearrangement that occurred between fungal communities in Gazi Bay and Mida Creek as a result of nutrient influx. We observed that fungal phylotypes mostly partitioned into different modules in both Gazi Bay and Mida Creek (Fig 5). This observation implies that the fungal communities comprised several modules that perform different ecological functions while each module is composed of diverse fungal species with similar adaptation and ecological function [19]. Niche partitioning within microbial communities promotes co-evolution and reduces negative associations among microbial phylotypes [59]. Meanwhile, the observation that a higher number of modules were detected in Gazi Bay implies that in Gazi Bay, the fungal communities were much more complex in terms of diversity of ecological functions and metabolic processes since each module is a separate niche with species that are involved in unique ecological roles [60]. This finding, in addition to the reported significantly higher fungal richness (Fig 1) and abundance of saprotrophs (wood, litter, soil and undefined saprotrophs) in Gazi Bay (Fig 6) demonstrate that the fungal communities in Gazi Bay were metabolically more diverse compared to those of Mida Creek.

*Agaricomycetes* and *Sordariomycetes* dominated (27%) the modules in Gazi Bay and Mida Creek. These fungal classes are both wood decay fungi [61, 62]; however, most *Agaricomycetes* species are capable of decomposing both the cellulosic and lignin components of wood [61] and are among the most enriched fungal classes in most unpolluted mangrove sediment [17, 63]. In both study sites, there were significant associations between fungal phylotypes and several physicochemical parameters. This observation underlines the importance of environmental factors on the assembly of the mangrove mycobiome. However, of more relevance is the observation that the mangrove chemical parameters played more important roles in the fungal community assembly of Mida creek, where there were detected among the network connectors. This, together with the higher percentage of negative associations in Mida creek may imply that changes in nutrient concentration resulting from anthropogenic disturbances are important drivers of microbial network structures. This is particularly important because mean values of the analysed physicochemical parameters were generally higher in Mida Creek than Gazi Bay (Table 1). Similarly, Sun et al. [64] reported that nutrient fluxes due to seasonal differences influenced the microbial network structures of a wastewater treatment plant. Overall, intercorrelating environmental factors and the mangrove species differentiation shaped the assembly patterns of the fungal communities in Gazi Bay and Mida Creek.

## Conclusion

In this study, we found that fungal community richness was lower in the anthropogenically disturbed Mida Creek, while species diversity was relatively unaffected. The overall alpha diversity was found to be co-influenced by differences in physicochemical parameters and mangrove species. The pattern of fungal community assembly in both sites of study was determined by a combination of mangrove species differences, geographical differences and alterations in physicochemical parameters. Nutrient increase in Mida Creek increased competition among fungal phylotypes. Also, our investigation of functional diversity suggests that the fungal communities in Gazi Bay were ecologically more diverse than the communities in Mida Creek. The overall findings from this study are important in developing policy guidelines for the protection of mangroves along the Kenya’s coast, particularly, considering the global climate change situation. Overall, the study objectives were achieved and we demonstrate that anthropogenic activities influenced fungal richness, community assembly and their ecological functions in the mangrove ecosystems investigated.

## Supporting information

S1 Fig Depth-based comparison of physicochemical parameters across the two sites. (TIF)

S2 Fig Within site comparison of physicochemical parameters according to depth of sampling in Gazi Bay. (TIF)

S3 Fig Within site comparison of physicochemical parameters according to depth of sampling in Mida Creek. (TIF)

S4 Fig Unique and shared ASVs across Gazi Bay and Mida Creek. (TIF)

S5 Fig Shared and unique ASVs across mangrove species in Gazi Bay and Mida Creek. (TIF)

S6 Fig Mangrove species to site comparison of ASVs. (TIF)

S7 Fig Distribution of fungi in Gazi Bay and Mida Creek at the phylum level taxonomic rank. (TIF)

S8 Fig Differentially abundant fungal genera across mangrove species (A) and across sites (B).
(TIF)

S1 Table Fungal alpha diversity and richness comparison for mangrove species in Gazi Bay and Mida Creek. (PDF)

S2 Table Pair-wise permutational multivariate analysis of variance based on site and mangrove species differentiation. (PDF)

S3 Table RDA variance explained by site differences and mangrove plant species. (PDF)

S4 Table Fungal relative abundance at the class and family taxonomic ranks. (XLSX)

S5 Table Network topological properties. (PDF)

## References

[1] Alongi DM. Carbon Cycling and Storage in Mangrove Forests. Ann Rev Mar Sci. 2014;6:195–219. doi: 10.1146/annurev-marine-010213-135020 24405426

[2] Zhang CJ, Pan J, Duan CH, Wang YM, Liu Y, Sun J, et al. Prokaryotic Diversity in Mangrove Sediments across Southeastern China Fundamentally Differs from That in Other Biomes. mSystems. 2019;4:1–15. doi: 10.1128/mSystems.00442-19 31506265 PMC6739103

[3] Arfi Y, Buée M, Marchand C, Levasseur A, Record E. Multiple markers pyrosequencing reveals highly diverse and host-specific fungal communities on the mangrove trees Avicennia marina and Rhizophora stylosa. FEMS Microbiol Ecol. 2012;79:433–444. doi: 10.1111/j.1574-6941.2011.01236.x 22092266

[4] Liu M, Huang H, Bao S, Tong Y. Microbial community structure of soils in Bamenwan mangrove wetland. Sci Rep. 2019;9:1–11. 31182804 10.1038/s41598-019-44788-x PMC6557889

[5] Holguin G, Vazquez P, Bashan Y. The role of sediment microorganisms in the productivity, conservation, and rehabilitation of mangrove ecosystems: An overview. Biol Fertil Soils. 2001;33:265–278.

[6] Simões MF, Antunes A, Ottoni CA, Amini MS, Alam I, Alzubaidy H, et al. Soil and Rhizosphere Associated Fungi in Gray Mangroves (Avicennia marina) from the Red Sea—A Metagenomic Approach. Genom Proteom Bioinform. 2015;13:310–320. doi: 10.1016/j.gpb.2015.07.002 26549842 PMC4678792

[7] Le Calvez T, Burgaud G, Mahé S, Barbier G, Vandenkoornhuyse P. Fungal diversity in deep-sea hydrothermal ecosystems. Appl Environ Microbiol. 2009;75:6415–6421. doi: 10.1128/AEM.00653-09 19633124 PMC2765129

[8] Anne KK, Romano KM, Remmy WK, Edward NK, Huxley MM, Hamadi IB. Diversity of fungi in sediments and water sampled from the hot springs of Lake Magadi and Little Magadi in Kenya. Afr J Microbiol Res. 2016;10:330–338.

[9] Liu P, Wang X, Li J, Qin W, Xiao C, Zhao X et al. Pyrosequencing Reveals Fungal Communities in the Rhizosphere of Xinjiang Jujube. Biomed Res Int. 2015:1–8. doi: 10.1155/2015/972481 25685820 PMC4313056

[10] Alongi DM. Bacterial productivity and microbial biomass in tropical mangrove sediments. Microb Ecol. 1988;15:59–79. doi: 10.1007/BF02012952 24202863

[11] Ghizelini AM, Mendonça-Hagler LCS, Macrae A. Microbial diversity in Brazilian mangrove sediments: a mini review. Braz J Microbiol. 2012;43:1242–1254. doi: 10.1590/S1517-83822012000400002 24031949 PMC3769006

[12] Booth JM, Fusi M, Marasco R, Michoud G, Fodelianakis S, Merlino G et al. The role of fungi in heterogeneous sediment microbial networks. Sci Rep. 2019;9:7537 doi: 10.1038/s41598-019-43980-3 31101834 PMC6525233

[13] Allard SM, Costa MT, Bulseco AN, Helfer V, Wilkins LGE, Hassenrück C et al. Introducing the Mangrove Microbiome Initiative: identifying microbial research priorities and approaches to better understand, protect, and rehabilitate mangrove ecosystems. mSystems.2020;5:e00658–20. doi: 10.1128/mSystems.00658-20 33082281 PMC7577295

[14] Lin X, Hetharua B, Lin L, Xu H, Zheng T, He Z, et al. Mangrove Sediment Microbiome: Adaptive Microbial Assemblages and Their Routed Biogeochemical Processes in Yunxiao Mangrove National Nature Reserve, China. Microb Ecol. 2019;78:57–69. doi: 10.1007/s00248-018-1261-6 30284602

[15] Muwawa EM, Obieze CC, Makonde HM, Jefwa JM, Kahindi JHP, Khasa DP. 16S rRNA gene amplicon-based metagenomic analysis of bacterial communities in the rhizospheres of selected mangrove species from Mida Creek and Gazi Bay, Kenya. PLOS ONE. 2021;16 (3):e0248485. doi: 10.1371/journal.pone.0248485 33755699 PMC7987175

[16] Jenoh EM, De Villiers EP, De Villiers SM, Okoth S, Jefwa J, Kioko E, et al. Infestation mechanisms of two woodborer species in the mangrove Sonneratia alba J. Smith in Kenya and co-occurring endophytic fungi. PLoS ONE. 2019;14:1–20. 10.1371/journal.pone.0221285 PMC6777984 31585459

[17] Haldar S, Nazareth SW. Diversity of fungi from mangrove sediments of Goa, India, obtained by metagenomic analysis using Illumina sequencing. 3 Biotech. 2019;9:1–5. 10.1007/s13205-019-1698-4 PMC6449407 30997301

[18] Sharma A, Lal R. Survey of (Meta)genomic Approaches for Understanding Microbial Community Dynamics. Indian J Microbiol. 2017;57(1):23–38. doi: 10.1007/s12088-016-0629-x 28148977 PMC5243251

[19] Xiong C, He J, Singh BK, Zhu Y, Wang J, Li P, et al. Rare taxa maintain the stability of crop mycobiomes and ecosystem functions. Environ Microbiol. 2020: doi: 10.1111/1462-2920.15262 32996254

[20] Hartman K, van der Heijden MGA, Wittwer RA, Banerjee S, Walser JC, Schlaeppi K. Cropping practices manipulate abundance patterns of root and soil microbiome members paving the way to smart farming. Microbiome. 2018;6:14. doi: 10.1186/s40168-017-0389-9 29338764 PMC5771023

[21] Obieze CC, Chikere CB, Selvarajan R, Adeleke R, Ntushelo K, Akaranta O. Functional attributes and response of bacterial communities to nature-based fertilization during hydrocarbon remediation. Int Biodeterior Biodegradation. 2020;154:105084.

[22] Lai J, Cheah W, Palaniveloo K, Suwa R, Sharma S. A Systematic Review of the Physicochemical and Microbial Diversity ofWell-Preserved, Restored, and DisturbedMangrove Forests: What Is Known and What Is the Way Forward? Forests 2022;13:2160.

[23] Lang’at JKS. Variability of mangrove forests along the Kenyan coast. Unpublished. 2008;(20).

[24] Owuor MA, Icely J, Newton A. Community perceptions of the status and threats facing mangroves of Mida Creek, Kenya: Implications for community based management. Ocean Coast Manag. 2019;175(March):172–9.

[25] Matthijs S, Tack J, van Speybroeck D, Koedam N. Mangrove species zonation and soil redox state, sulphide concentration and salinity in Gazi Bay (Kenya), a preliminary study. Mangroves Salt Marshes. 1999;3(4):243–9.

[26] Gwada P, Kairo JG. Litter production in three mangrove stands of Mida Creek, Kenya. South African J Bot. 2001;67(3):443–9.

[27] Dahdouh-Guebas F, Van Pottelbergh I, Kairo JG, Cannicci S, Koedam N. Human-impacted mangroves in Gazi (Kenya): Predicting future vegetation based on retrospective remote sensing, social surveys, and tree distribution. Mar Ecol Prog Ser. 2004;272:77–92.

[28] Wu P, Xiong X, Xu Z, Lu C, Cheng H, Lyu X, et al. Bacterial communities in the rhizospheres of three mangrove tree species from Beilun Estuary, China. PLOS ONE. 2016;11(10):1–13. doi: 10.1371/journal.pone.0164082 27695084 PMC5047532

[29] Giannopoulos G, Lee DY, Neubauer SC, Brown BL, Franklin RB. A simple and effective sampler to collect undisturbed cores from tidal marhes. 2019;

[30] Brupbacher RH, Bonner WP, Sedberry J Jr. Analytical methods and procedures used in the soil testing laboratory. 1968;15.

[31] Tedersoo L, Bahram M, Põlme S, Kõljalg U, Yorou NS, Wijesundera R, et al. Global diversity and geography of soil fungi. Science. 2014:346. 10.1126/science.1256688 25430773

[32] Bolyen E, Rideout JR, Dillon MR, Bokulich NA, Abnet CC, Al-Ghalith GA, et al. Reproducible, interactive, scalable and extensible microbiome data science using QIIME 2. Vol. 37, Nature Biotechnology. 2019. 10.1038/s41587-019-0209-9 PMC7015180 31341288

[33] Callahan BJ, McMurdie PJ, Rosen MJ, Han AW, Johnson AJA, Holmes SP. DADA2: High-resolution sample inference from Illumina amplicon data. Nat Methods. 2016;13(7):581–3. doi: 10.1038/nmeth.3869 27214047 PMC4927377

[34] R Core Team, R: A Language and Environment for Statistical Computing. R Foundation for Statistical Computing, Vienna, Austria. 2019. https://www.R-project.org/

[35] de Mendiburu F. agricolae: Statistical Procedures for Agricultural Research. 2020.

[36] Segata N, Izard J, Waldron L, Gevers D, Miropolsky L, Garrett WS, et al. Metagenomic biomarker discovery and explanation. Genome Biol. 2011 Jun 24;12(6):1–18. doi: 10.1186/gb-2011-12-6-r60 21702898 PMC3218848

[37] Breiman L. Random forests. Mach Learn. 2001;45:5–32.

[38] Dhariwal A, Chong J, Habib S, King LI, Agellon BL, Xia J. MicrobiomeAnalyst: a web-based tool for comprehensive statistical, visual and meta-analysis of microbiome data. Nucleic Acids Res. 2017;45:180–188. doi: 10.1093/nar/gkx295 28449106 PMC5570177

[39] Nguyen NH, Song Z, Bates ST, Branco S, Tedersoo L, Menke J, et al. FUNGuild: An open annotation tool for parsing fungal community datasets by ecological guild. 2016. 10.1016/j.funeco.2015.06.006.

[40] Faust K, Raes J. CoNet app: inference of biological association networks using Cytoscape. F1000Research. 2016;5:1519. doi: 10.12688/f1000research.9050.2 27853510 PMC5089131

[41] Blondel VD, Guillaume JL, Lambiotte R, Lefebvre E. Fast unfolding of communities in large networks. J Stat Mech. 2008:P10008.

[42] Guimera R, Amaral LAN. Functional cartography of complex metabolic networks. Nature. 2005;433(7028):895–900. doi: 10.1038/nature03288 15729348 PMC2175124

[43] Krüger C, Kohout P, Janoušková M, Püschel D, Frouz J, Rydlová J. Plant communities rather than soil properties structure arbuscular mycorrhizal fungal communities along primary succession on a mine spoil. Front Microbiol. 2017 Apr 20;8(APR). doi: 10.3389/fmicb.2017.00719 28473828 PMC5397529

[44] Nathan VK, Vijayan J, Ammini P. Comparison of bacterial diversity from two mangrove ecosystems from India through metagenomic sequencing: Comparative mangrove bacterial diversity using metagenomics. Reg Stud Mar Sci. 2020;35:101184.

[45] Zhu P, Wang Y, Shi T, Huang G, Gong J. Genetic Diversity of Benthic Microbial Eukaryotes in Response to Spatial Heterogeneity of Sediment Geochemistry in a Mangrove Ecosystem. Estuaries Coast. 2018;41:751–764.

[46] Cheung MK, Wong CK, Chu KH, Kwan HS. Community Structure, Dynamics and Interactions of Bacteria, Archaea and Fungi in Subtropical Coastal Wetland Sediments. Sci Rep. 2018;8:14397. doi: 10.1038/s41598-018-32529-5 30258074 PMC6158284

[47] Bahram M, Peay KG, Tedersoo L. Local-scale biogeography and spatiotemporal variability in communities of mycorrhizal fungi. New Phytol. 2015;205:1454–1463. doi: 10.1111/nph.13206 25767850

[48] Sanka Loganathachetti D, Poosakkannu A, Muthuraman S. Fungal community assemblage of different soil compartments in mangrove ecosystem. Sci Rep. 2017;7:1–9. 28819270 10.1038/s41598-017-09281-3 PMC5561109

[49] Zhang Y, Yang Q, Ling J, Van Nostrand JD, Shi Z, Zhou J, et al. Diversity and structure of diazotrophic communities in mangrove rhizosphere, revealed by high-throughput sequencing. Front Microbiol. 2017;8:1–11. 29093705 10.3389/fmicb.2017.02032 PMC5651520

[50] Purahong W, Sadubsarn D, Tanunchai B, Wahdan SFM, Sansupa C, Noll M, et al. First insights into the microbiome of a mangrove tree reveal significant differences in taxonomic and functional composition among plant and soil compartments. Microorganisms. 2019;7(12). doi: 10.3390/microorganisms7120585 31756976 PMC6955992

[51] Velez P, González MC, Capello-García S, Rosique-Gil E, Hanlin RT. Diversity of marine ascomycetes from the disturbed sandy beaches of Tabasco, Mexico. J Mar Biolog Assoc UK. 2015;95:897–903.

[52] Zhang T, Wang NF, Zhang YQ, Liu HY, Yu LY. Diversity and distribution of fungal communities in the marine sediments of Kongsfjorden, Svalbard (High Arctic). Sci Rep. 2015;5:1–11. doi: 10.1038/srep14524 26494429 PMC4615975

[53] Li W, Wang MM, Wang XG, Cheng XL, Guo JJ, Bian XM, et al. Fungal communities in sediments of subtropical Chinese seas as estimated by DNA metabarcoding. Sci Rep. 2016;6:1–9. 27198490 10.1038/srep26528 PMC4873734

[54] Devadatha B, Jones EBG, Pang KL, Abdel-Wahab MA, Hyde KD, Sakayaroj J, et al. Occurrence and geographical distribution of mangrove fungi. Fungal Divers. 2021;106:137–227.

[55] Jie MZ, Jiang LC, Di YSD, Crews P. The Bioactive Secondary Metabolites from Talaromyces species. Nat Prod Bioprospect. 2016;6:1–24. doi: 10.1007/s13659-015-0081-3 26746215 PMC4749520

[56] Thorati M, Mishra JK, Kumar S. Isolation, Identification of Endophytic Fungi from Mangrove Roots along the Coast of South Andaman Sea, Andaman and Nicobar Islands,India. Oceanogr Mar Biol. 2016;5:1–6.

[57] Tomsheck RA, Strobel AG, Booth E, Geary B, Spakowicz D, Knighton B, et al. Hypoxylon sp., an Endophyte of Persea indica, Producing 1,8-Cineole and Other Bioactive Volatiles with Fuel Potential. Microb Ecol. 2010; 60:903–914 doi: 10.1007/s00248-010-9759-6 20953951

[58] Le TMH, Do TQ, Doan THM, Vu TQ, Nguyen AM, Vu THT et al. Chemical Composition and Biological Activities of Metabolites from the Marine Fungi Penicillium sp. Isolated from Sediments of Co To Island, Vietnam. Molecules. 2019;24 (3830): 1–11. doi: 10.3390/molecules24213830 31652901 PMC6864758

[59] Bauer MA, Kainz K, Carmona-Gutierrez D, Madeo F. Microbial wars: Competition in ecological niches and within the microbiome. Microb Cell. 2018;5:215–219. doi: 10.15698/mic2018.05.628 29796386 PMC5961915

[60] Jiao S, Xu Y, Zhang J, Hao X, Lu Y. Core Microbiota in Agricultural Soils and Their Potential Associations with Nutrient Cycling. mSystems 2019;4: doi: 10.1128/mSystems.00313-18 30944882 PMC6435817

[61] Lundell TK, Mäkelä MR, de Vries RP, Hildén KS. Genomics, lifestyles and future prospects of wood-decay and litter-decomposing basidiomycota. In: Advances in Botanical Research. Academic Press Inc. 2014:329–370.

[62] Jones EBG, Devadatha B, Abdel-Wahab MA, Dayarathne MC, Zhang SN, Hyde KD, et al. Phylogeny of new marine Dothideomycetes and Sordariomycetes from mangroves and deep-sea sediments. Botanica Marina. 2020;63:155–181.

[63] Lee NLY, Huang D, Quek ZBR, Lee JN, Wainwright BJ. Mangrove-Associated Fungal Communities Are Differentiated by Geographic Location and Host Structure. Front Microbiol. 2019;10:2456. doi: 10.3389/fmicb.2019.02456 31736902 PMC6831645

[64] Sun C, Zhang B, Ning D, Zhang Y, Dai T, Wu L, et al. Seasonal dynamics of the microbial community in two full-scale wastewater treatment plants: Diversity, composition, phylogenetic group based assembly and co-occurrence pattern. *Water Res*. 2021;200:117295. doi: 10.1016/j.watres.2021.117295 34091223

