# Supplementary figures and images for: Diversity and assembly patterns of mangrove rhizosphere mycobiome along the Coast of Gazi Bay and Mida Creek in Kenya
Source: PLoS One. 2024 Apr 18;19(4):e0298237. doi: 10.1371/journal.pone.0298237 (PMC11025898; doi:10.1371/journal.pone.0298237)

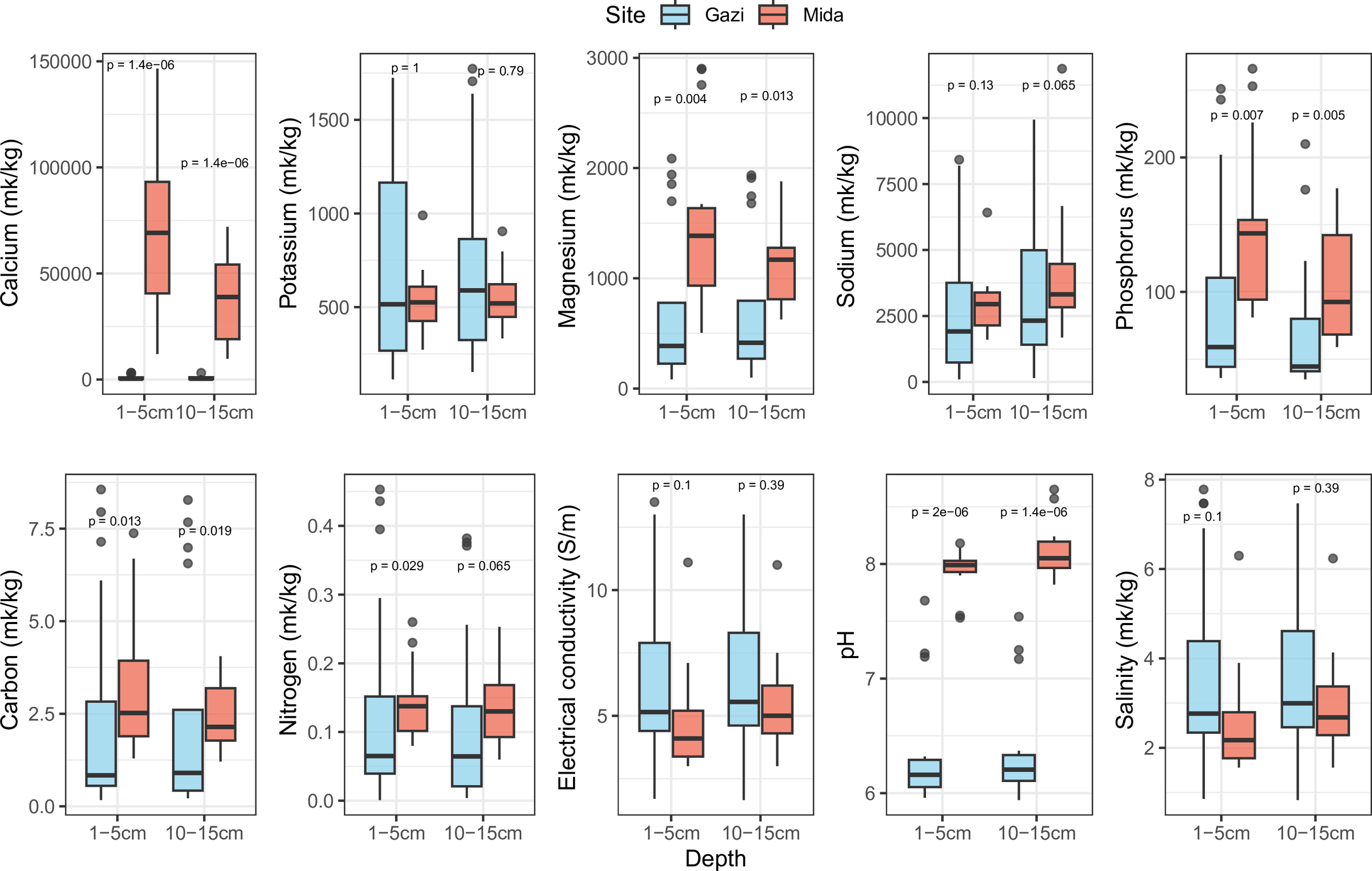

Supplement: S1 Fig — (TIF) [file pone.0298237.s001.tif]

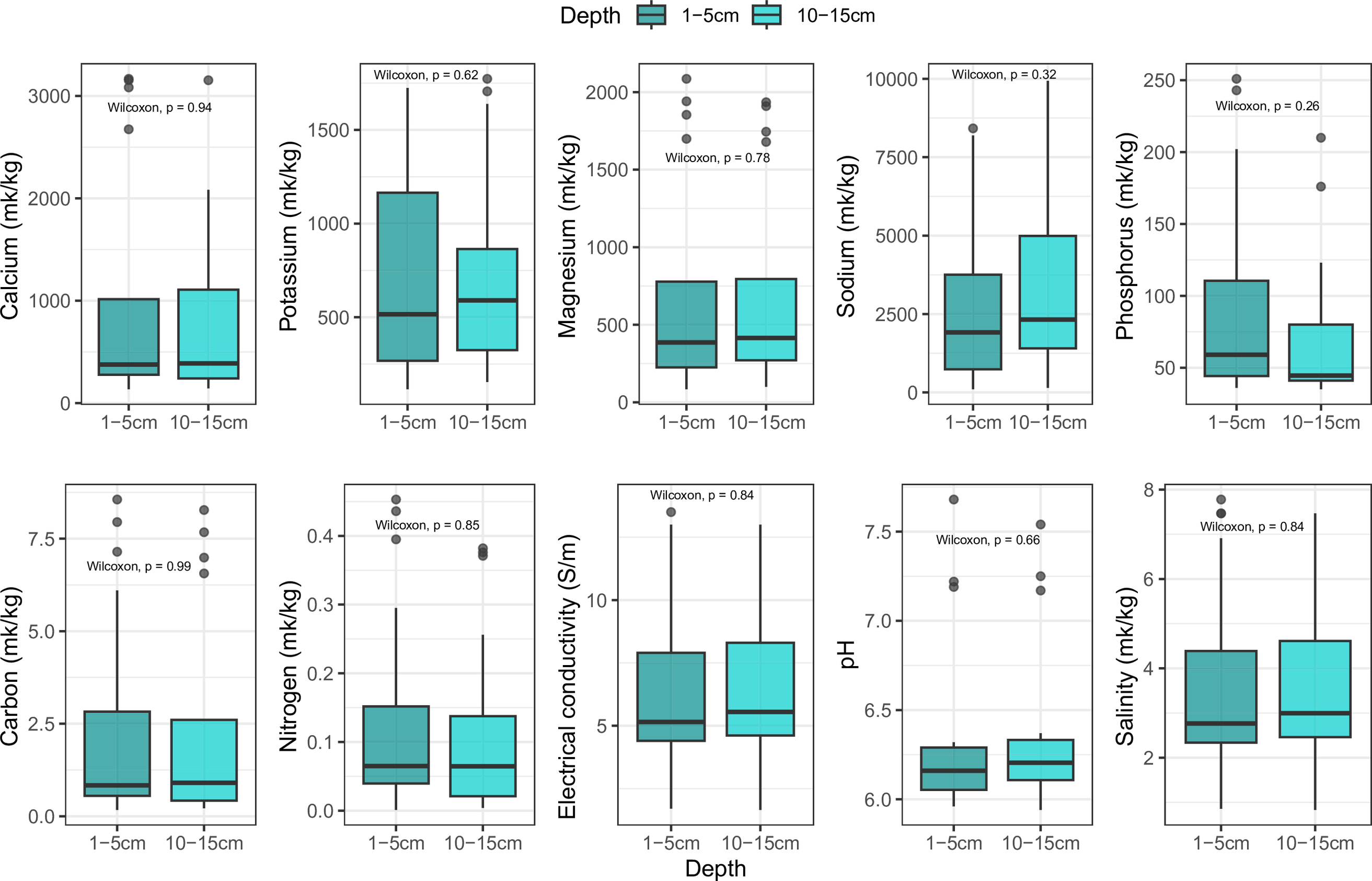

Supplement: S2 Fig — (TIF) [file pone.0298237.s002.tif]

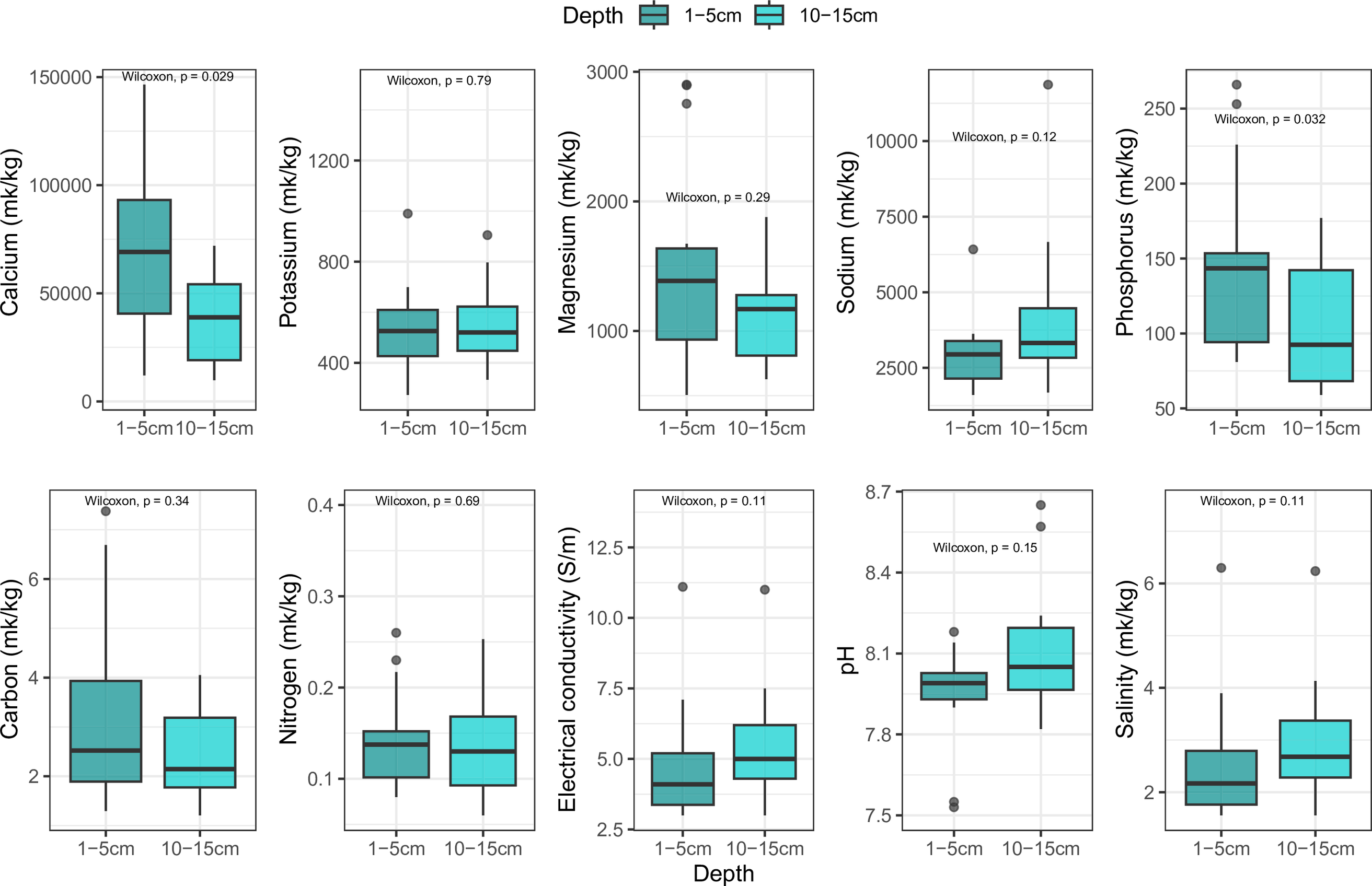

Supplement: S3 Fig — (TIF) [file pone.0298237.s003.tif]

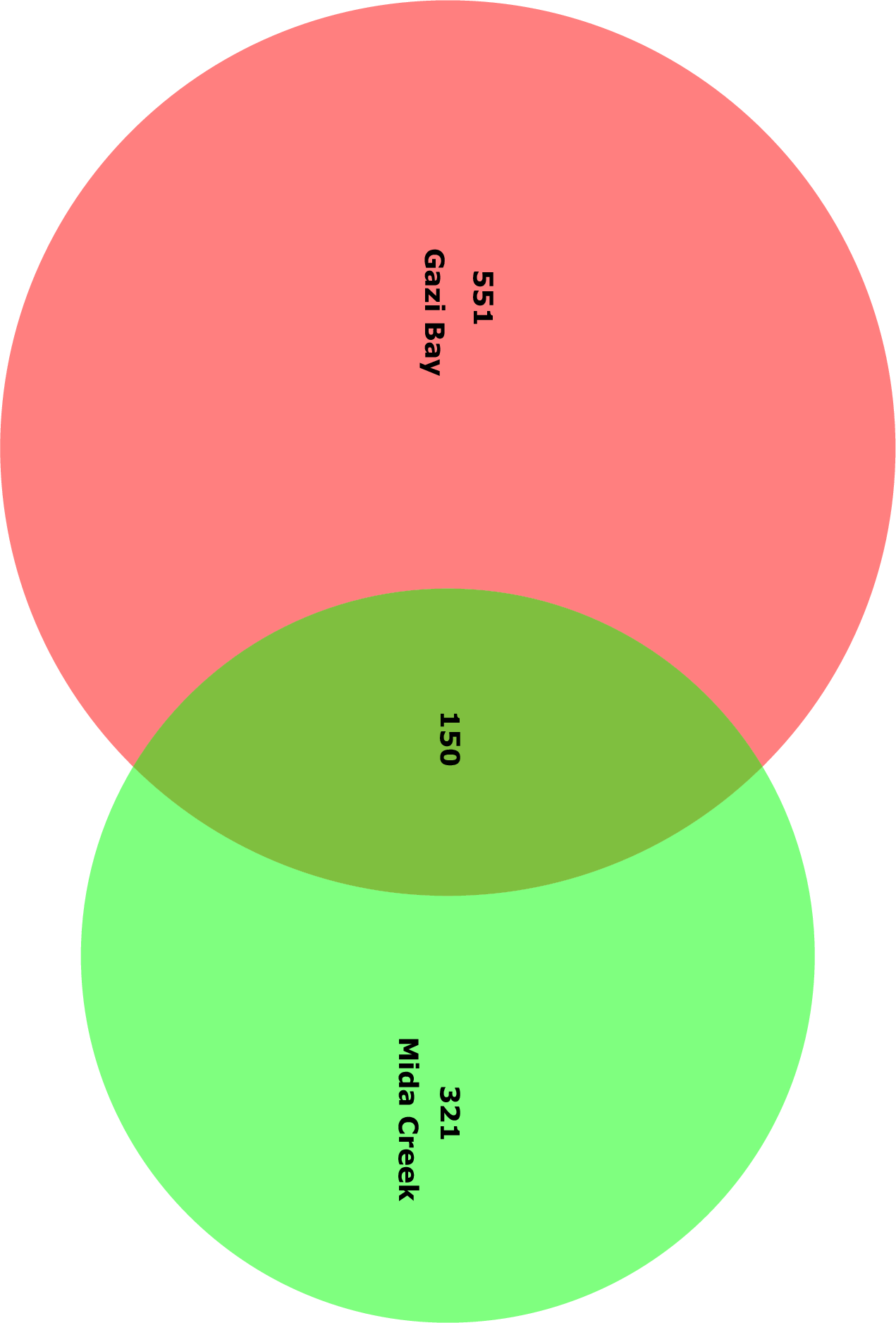

Supplement: S4 Fig — (TIF) [file pone.0298237.s004.tif]

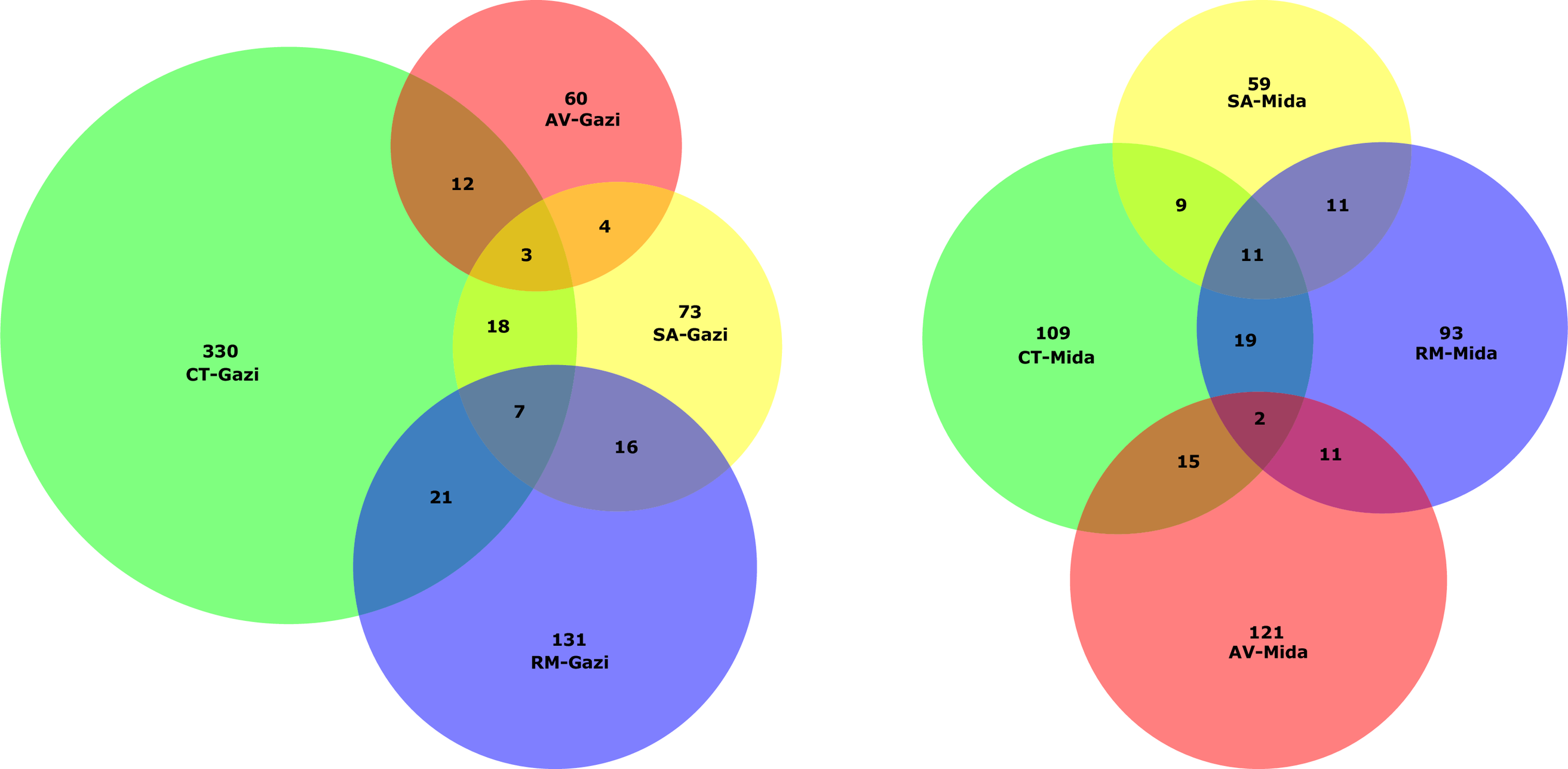

Supplement: S5 Fig — (TIF) [file pone.0298237.s005.tif]

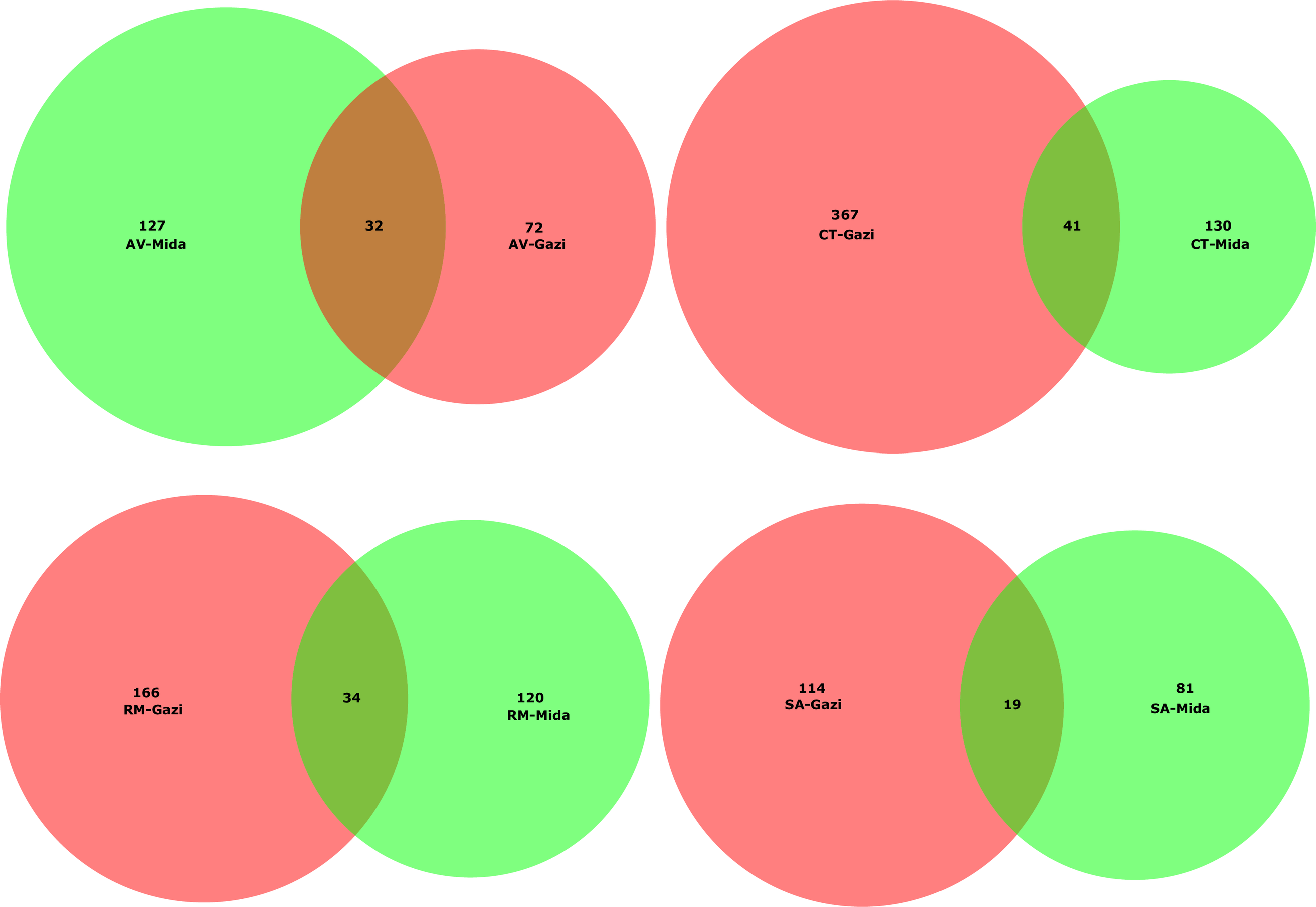

Supplement: S6 Fig — (TIF) [file pone.0298237.s006.tif]

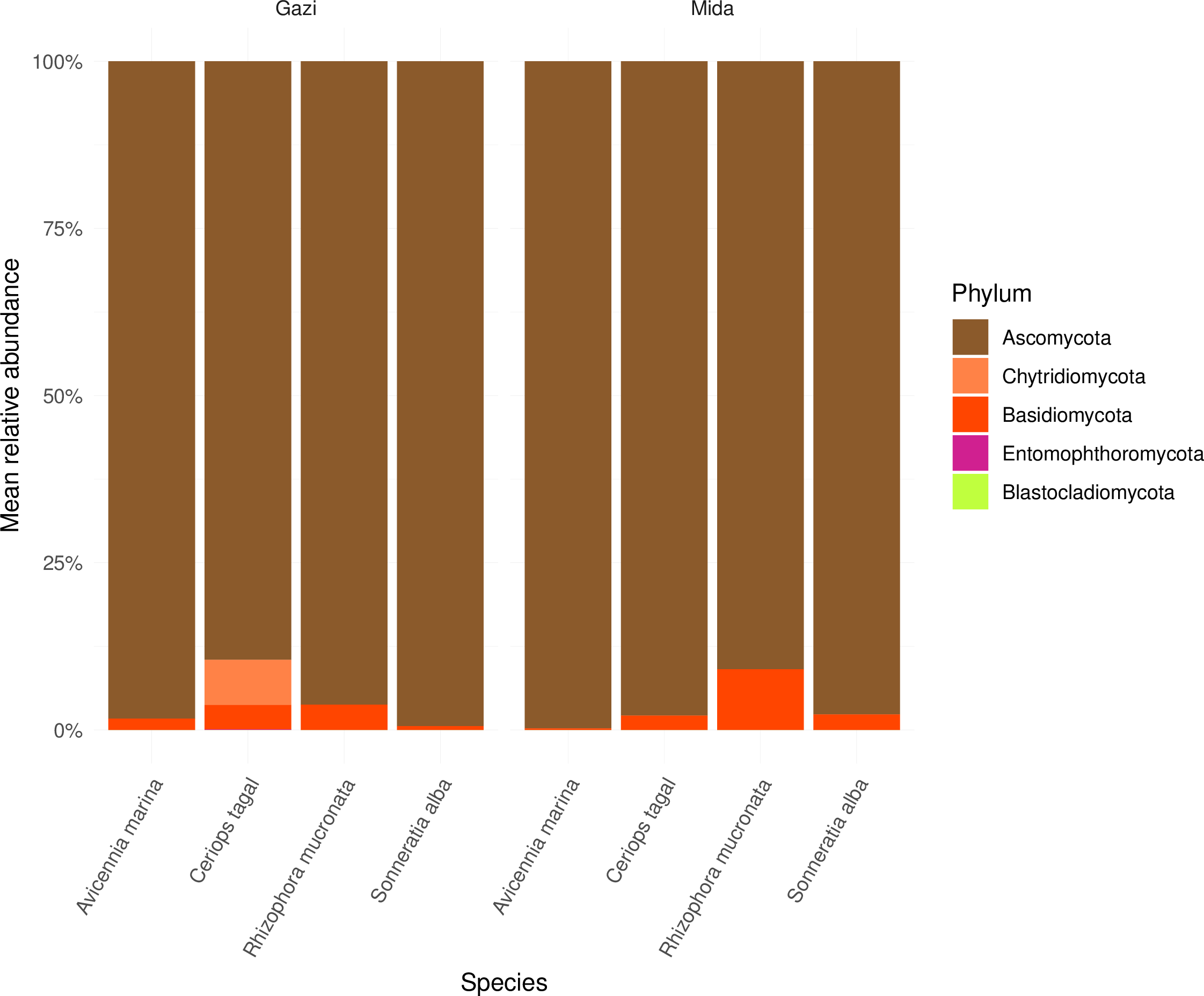

Supplement: S7 Fig — (TIF) [file pone.0298237.s007.tif]

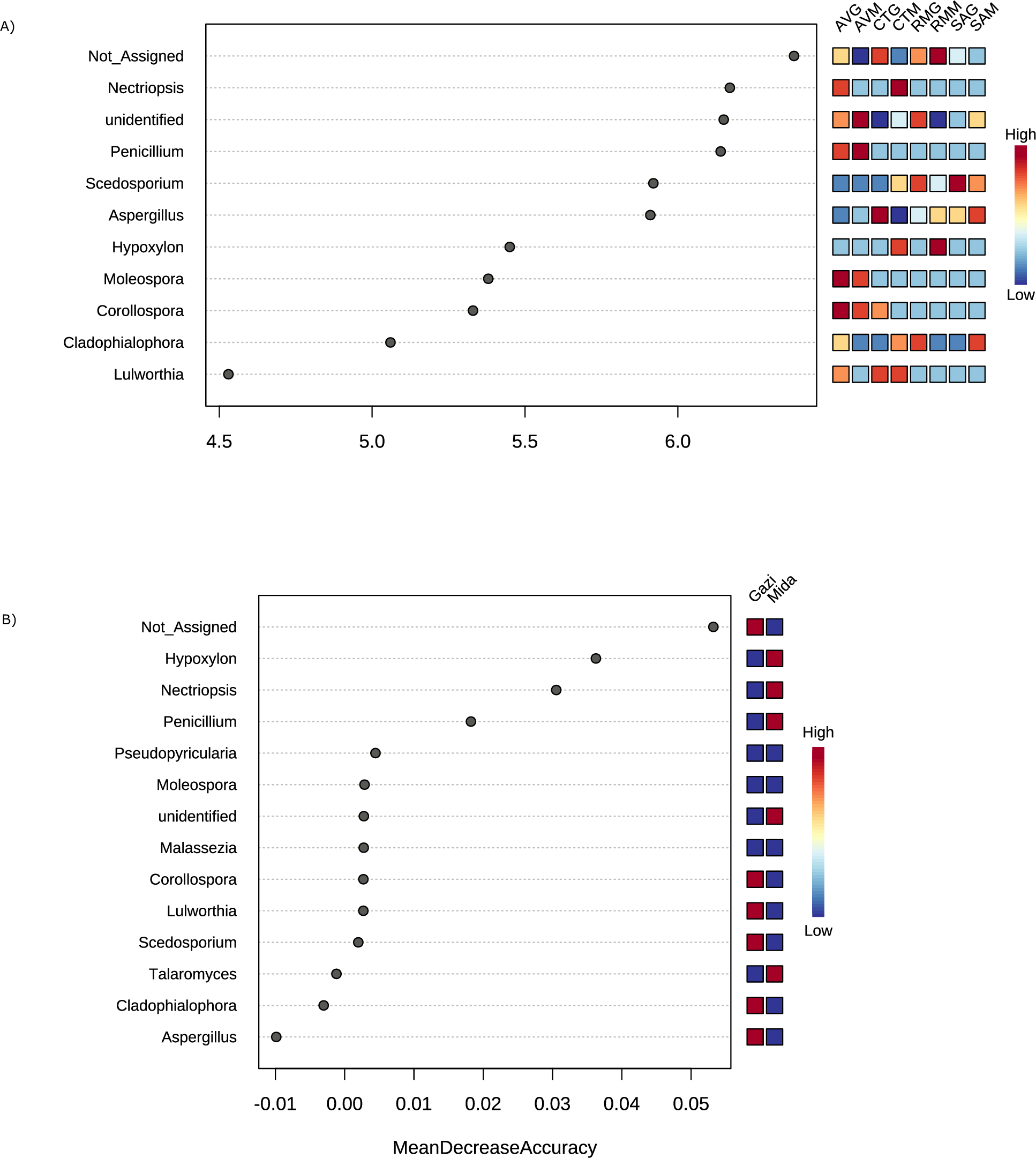

Supplement: S8 Fig — Differentially abundant fungal genera across mangrove species (A) and across sites (B). (TIF) [file pone.0298237.s008.tif]
