# Supplementary material for: Diversity and assembly patterns of mangrove rhizosphere mycobiome along the Coast of Gazi Bay and Mida Creek in Kenya
Source: PLoS One. 2024 Apr 18;19(4):e0298237. doi: 10.1371/journal.pone.0298237 (PMC11025898; doi:10.1371/journal.pone.0298237)
Supplement: S1 Table — (PDF) [file pone.0298237.s009.pdf]

S1 Table: Fungal alpha diversity and richness comparison for mangrove species in Gazi Bay and Mida Creek

**Gazi Bay**							
		Observed ASVs		Shannon entropy		Pielou's evenness	
Group1	Group2	p-value	q-value	p-value	q-value	p-value	q-value
AVG	CTG	0.001	0.018	0.009	0.048	0.074	0.208
	RMG	0.002	0.018	0.001	0.023	0.003	0.046
	SAG	0.746	0.860	0.699	0.752	0.606	0.707
CTG	RMG	0.128	0.298	0.248	0.408	0.021	0.097
	SAG	0.002	0.018	0.028	0.088	0.197	0.351
RMG	SAG	0.004	0.022	0.005	0.032	0.010	0.080
**Mida Creek**							
Group1	Group2	p-value	q-value	p-value	q-value	p-value	q-value
AVM	CTM	0.332	0.495	0.606	0.707	0.366	0.488
	RMM	0.799	0.860	0.174	0.348	0.174	0.348
	SAM	0.682	0.831	0.540	0.658	0.414	0.504
CTM	RMM	0.453	0.576	0.201	0.351	0.201	0.351
	SAM	0.435	0.576	0.197	0.351	0.071	0.208
RMM	SAM	1.000	1.000	0.289	0.426	0.289	0.445

[1]

[1]AVG - *A. marina* from Gazi, AVM - *A. marina* from Mida Creek, CTG - *C. tagal* from Gazi, CTM - *C. tagal* from Mida Creek, RMG - *R. mucronata* from Gazi, RMM - *R. mucronata* from Mida Creek, SAG - *S. alba* from Gazi, SAM - *S. alba* from Mida Creek.
