# Supplementary material for: Diversity and assembly patterns of mangrove rhizosphere mycobiome along the Coast of Gazi Bay and Mida Creek in Kenya
Source: PLoS One. 2024 Apr 18;19(4):e0298237. doi: 10.1371/journal.pone.0298237 (PMC11025898; doi:10.1371/journal.pone.0298237)
Supplement: S2 Table — (PDF) [file pone.0298237.s010.pdf]

S2 Table: Pair-wise permutational multivariate analysis of variance based on site and mangrove species differentiation.

Group 1	Group 2	Sample size	Permutations	pseudo-F	p-value	q-value
AVG	AVM	16	999	5.45377	0.002	0.0035
AVG	CTG	16	999	8.346152	0.001	0.002333
AVG	CTM	14	999	13.77533	0.001	0.002333
AVG	RMG	16	999	9.043074	0.001	0.002333
AVG	RMM	12	999	6.503388	0.002	0.0035
AVG	SAG	14	999	7.120572	0.001	0.002333
AVG	SAM	11	999	6.722958	0.007	0.008522
AVM	CTG	16	999	6.581798	0.001	0.002333
AVM	CTM	14	999	8.885845	0.002	0.0035
AVM	RMG	16	999	6.544891	0.001	0.002333
AVM	RMM	12	999	4.22796	0.004	0.005895
AVM	SAG	14	999	4.771604	0.001	0.002333
AVM	SAM	11	999	4.153275	0.011	0.01232
CTG	CTM	14	999	8.082426	0.001	0.002333
CTG	RMG	16	999	5.497838	0.001	0.002333
CTG	RMM	12	999	3.494835	0.005	0.006667
CTG	SAG	14	999	4.15466	0.001	0.002333
CTG	SAM	11	999	3.700572	0.006	0.007636
CTM	RMG	14	999	6.208459	0.001	0.002333
CTM	RMM	10	999	5.111877	0.003	0.004667
CTM	SAG	12	999	5.433585	0.003	0.004667
CTM	SAM	9	999	5.473427	0.015	0.015556
RMG	RMM	12	999	2.8034	0.002	0.0035
RMG	SAG	14	999	3.906734	0.001	0.002333
RMG	SAM	11	999	3.325058	0.005	0.006667
RMM	SAG	10	999	2.434757	0.012	0.012923
RMM	SAM	7	999	2.094173	0.079	0.079
SAG	SAM	9	999	2.50743	0.011	0.01232

[1]

[1] AVG - *A. marina* from Gazi, AVM - *A. marina* from Mida Creek, CTG - *C. tagal* from Gazi, CTM - *C. tagal* from Mida Creek, RMG - *R. mucronata* from Gazi, RMM - *R. mucronata* from Mida Creek, SAG - *S. alba* from Gazi, SAM - *S. alba* from Mida Creek.
