# Supplementary material for: Diversity and assembly patterns of mangrove rhizosphere mycobiome along the Coast of Gazi Bay and Mida Creek in Kenya
Source: PLoS One. 2024 Apr 18;19(4):e0298237. doi: 10.1371/journal.pone.0298237 (PMC11025898; doi:10.1371/journal.pone.0298237)
Supplement: S3 Table — (PDF) [file pone.0298237.s011.pdf]

S3 Table:

	Inertia	Proportion	Rank			
Total	0.9062	1				
Constrained	0.3450	0.3807	7			
Unconstrained	0.5612	0.6193	56			
Eigenvalues for constrained axes:						
RDA1	RDA2	RDA3	RDA4	RDA5	RDA6	RDA7
0.1042	0.0568	0.0461	0.04068	0.03801	0.03282	0.02637

*Inertia is variance
