# Supplementary material for: Diversity and assembly patterns of mangrove rhizosphere mycobiome along the Coast of Gazi Bay and Mida Creek in Kenya
Source: PLoS One. 2024 Apr 18;19(4):e0298237. doi: 10.1371/journal.pone.0298237 (PMC11025898; doi:10.1371/journal.pone.0298237)
Supplement: S5 Table — (PDF) [file pone.0298237.s013.pdf]

S5 Table: Network topological properties

Parameters	Gazi Bay	Mida Creek
Nodes	72	64
Edges	239	151
Positive interaction	183	50
Negative interaction	56	101
Average weighted degree	6.65	4.81
Network diameter	6	6
Network density	0.09	0.08
Modularity	0.54	0.45
Clustering coefficient	0.35	0.05
Average path length	2.75	2.79
